# Investigating the Influence of Climate Changes on Rodent Communities at a Regional-Scale (MIS 1-3, Southwestern France)

**DOI:** 10.1371/journal.pone.0145600

**Published:** 2016-01-20

**Authors:** Aurélien Royer, Sophie Montuire, Serge Legendre, Emmanuel Discamps, Marcel Jeannet, Christophe Lécuyer

**Affiliations:** 1 Université de Bordeaux, CNRS, MCC, PACEA, UMR 5199 F—33615 Pessac, France; 2 Université Lyon 1 et Ecole Normale Supérieure de Lyon, Laboratoire de Géologie de Lyon, UMR CNRS 5276, 69622 Villeurbanne, France; 3 Université de Bourgogne, Biogéosciences, UMR CNRS 6282, 6 Boulevard Gabriel, 21000 Dijon, France; 4 University of Bergen, AHKR Institute, Øysteinsgate 1, PO Box 7805, N-5020 Bergen, Norway; 5 Aix Marseille Université, LAMPEA, UMR CNRS 7269, MMSH, 5 rue du Château de l’Horloge, BP 647, 13094 Aix-en-Provence cedex 2, France; 6 Ecole Pratique des Hautes Etudes, Laboratoire EPHE PALEVO, 21000 Dijon, France; 7 Institut Universitaire de France, Paris, France; NYIT College of Osteopathic Medicine, UNITED STATES

## Abstract

Terrestrial ecosystems have continuously evolved throughout the Late Pleistocene and the Holocene, deeply affected by both progressive environmental and climatic modifications, as well as by abrupt and large climatic changes such as the Heinrich or Dansgaard-Oeschger events. Yet, the impacts of these different events on terrestrial mammalian communities are poorly known, as is the role played by potential refugia on geographical species distributions. This study examines community changes in rodents of southwestern France between 50 and 10 ky BP by integrating 94 dated faunal assemblages coming from 37 archaeological sites. This work reveals that faunal distributions were modified in response to abrupt and brief climatic events, such as Heinrich events, without actually modifying the rodent community on a regional scale. However, the succession of events which operated between the Late Pleistocene and the Holocene gradually led to establishing a new rodent community at the regional scale, with intermediate communities occurring between the Bølling and the Allerød.

## Introduction

Over the last glacial period (115.0 to 11.7 ky BP) climates have regularly oscillated, varying from mild periods called Greenland Interstadials (GI) to cold periods called Greenland Stadials (GS), as well as to arid and cold periods such as Heinrich events. These climatic oscillations were imprinted in the Greenland ice-core records, allowing the estimation of duration of these climatic stages to be between 500 and 2,000 years [1]. The shifts between cold and warm phases were fast, with most of the climatic change being accomplished within 10 to 200 years, and are characterized by mean annual temperature changes comprised between 8 and 15°C in Greenland [1,2,3]. These climatic oscillations have been recorded in a large range of marine and terrestrial environments worldwide. They have deeply influenced the mid-latitude terrestrial ecosystems, as suggested by the observed variations of pollen assemblages from marine and terrestrial continuous deposits [4,5,6,7,8]. These changes would probably also have deeply influenced faunal communities and hominid populations. Unfortunately, the responses of mammal communities to these events are poorly known as the discontinuous nature of continental deposits and their frequent lack of a faunal content of biostratigraphic interest frequently generates datasets of poor temporal resolution that preclude adequate reconstructions and inter-proxy comparisons.

The aim of this paper is to investigate the evolution of small mammal communities from southwestern France between 50 and 10 ky BP, by integrating both biotic and abiotic (geographical) factors that shaped their structures. Southwestern France was exposed to successive climatic events as recorded in speleothems, floral, and faunal assemblages [6,9,10,11,12]. Geographically this region is a well-defined basin characterized by an abundance of archaeological sequences rich in faunal remains. This continental area, located at the western end of Europe, is limited westward by the Atlantic Ocean, southward by the Pyrenean Mountains, and northeastward by the Massif Central Mountains. These physical boundaries (albeit permeable) influenced the patterns of faunal evolution, especially the capacity of species to migrate or to mix with allochthonous populations. Rodent associations are widely used to reconstruct Quaternary climates and their environments at both the local (e.g. [13,14,15,16]) and regional scales (e.g. [17,18,19]), because it is generally considered that their small size, their high rates of reproduction, their restricted habitat, and their absence of migratory behavior make them reliable indicators of environmental changes [14,20,21,22,23]. Interestingly, this region has a long tradition of archaeological and palaeontological investigation and curation of faunal remains in museums since the middle of the XIXth century, which led to many studies of small mammal fossils (e.g. [24,25,26,27,28,29,30,31,32,33,34,35]). This paper focuses on impacts of climatic changes on the evolution and dynamics of rodent communities at both local and regional scales of analysis, thus offering the possibility of investigating climatic and ecological processes that contributed to structure the past and present communities.

## Materials and Methods

### Ethics Statement

**Fig 1:**
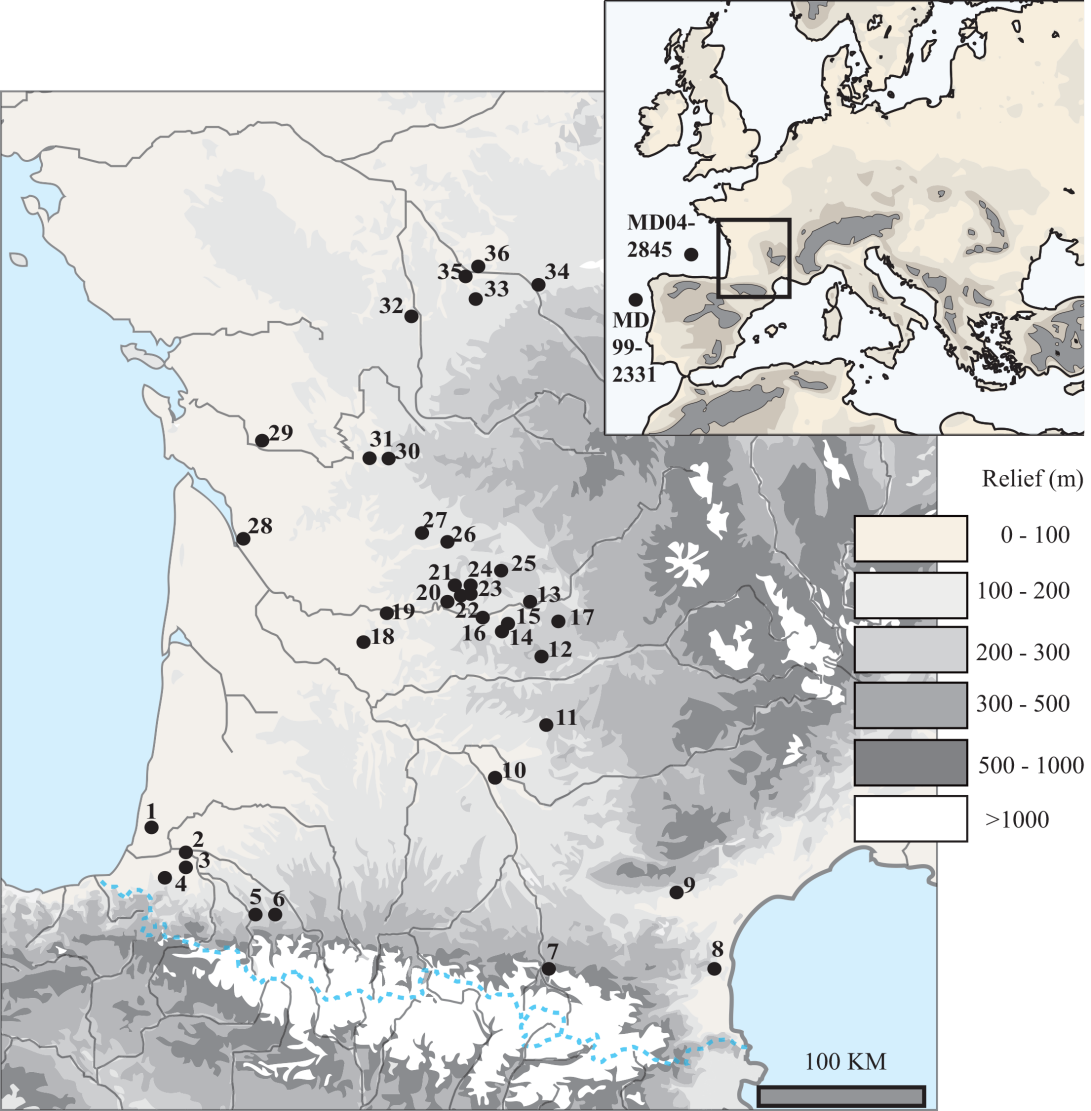
Geographic map with the location of the archeological sites used in this study along with the location of the deep-sea cores whose sedimentary record helped to establish the paleoclimatic correlations (MD04-2845 and MD99-2331). Numbers identify the archeological sites as follows: 1-Dufaure. 2-Grotte Duruthy. 3-Bourrouilla. 4- Isturitz. 5-Espélugues. 6-Bois du Cantet. 7-La Vache. 8-Conques. 9-Abeurador. 10-Grotte du Salpêtre de Pompignan. 11-Abri Fontalès. 12-Peyrugues. 13-Peyrazet. 14-Roc de Combe. 15-Le Piage. 16-Combe Grenal. 17-Les Fieux. 18-Borie del Rey. 19-Le Flageolet 1 and 2. 20-Moulin du Roc. 21-La Ferrassie. 22-Abri Pataud. 23-Grotte Comarque. 24-Abri du Facteur. 25-Castanet. 26-Combe Saunière. 27-Pont d’Ambon. 28-Roc de Marcamps. 29-La Roche à Pierrot. 30-Montgaudier (Abri Pintaud-Gaudry). 31-Abri Ragout. 32-Bois Ragot. 33-Taillis-des-Coteaux. 34-Abri Garenne. 35-Les Cottés. 36-Abri Fritsch.

No permits were required for the described study, which complied with all relevant regulations. The largest part of the dataset comes from previously published analyses of rodent remains. Only data from two sites are still unpublished. Data from these site were collected by the first author (A.R.) and are accessible in his PhD thesis [36]. The information for all sites and previously published work is detailed in Table 1 and the supplementary tables with each site’s location displayed in Fig 1. The collection of the samples was approved by the directors of archaeological excavations. The remains (n>400,000) are classified by location and archaeological stratigraphy and are deposited in the University of Bordeaux, but there are no ID numbers. They are in good condition and are associated with all information needed in order to be published, retrieved and/or analyzed by others. Collection sites are both public and private. We also confirm that we had permits for the work associated with the doctoral thesis.

**Table 1:** Presence (1) and absence (0) of some rodent species retrieved from the studied archaeological units with the total Minimum Number of Individuals (MNI) when provided in the original reference.

Site number of Fig 1	Site	Stratigraphic units	*Microtus oeconomus*	*Microtus arvalis-agrestis*	*Microtus gregalis*	*Microtus (T*.*) subterraneus*	*Microtus (T*.*) duodecimcostatus*	*Microtus (T*.*) multiplex*	*Clethrionomys glareolus*	*Spermophilus* sp.	*Dicrostonyx torquatus*	*Chionomys nivalis*	*Arvicola amphibius*	*Arvicola sapidus*	*Apodemus sylvaticus*	*Eliomys quercinus*	*Glis glis*	*Cricetus cricetus*	*Sicista betulina*	References	MNI
	**Present-day**		0	1	0	0	1	0	1	0	0	1	1	1	1	1	1	0	0	[37]	-
**1**	**Dufaure**	3	1	1	0	0	0	0	0	0	0	0	1	0	1	0	0	0	0	[38]	10
**1**	**Dufaure**	4	1	1	0	0	0	0	0	0	0	0	1	0	1	0	0	0	0	[38]	301
**1**	**Dufaure**	5	1	1	0	0	0	0	0	0	0	0	1	0	1	0	0	0	0	[38]	378
**1**	**Dufaure**	6	1	1	0	0	0	0	0	0	0	0	1	0	1	0	0	0	0	[38]	78
**2**	**La grotte Duruthy**	C3	0	1	0	0	0	0	0	0	0	0	1	0	1	0	0	0	0	[28]	5
**2**	**La grotte Duruthy**	C4	0	1	0	0	0	0	0	0	0	0	1	0	0	0	0	0	0	[28]	3
**2**	**La grotte Duruthy**	C5	1	1	0	0	0	0	0	0	0	0	1	0	0	0	0	0	0	[28]	5
**3**	**Bourrouilla**	b2	1	1	0	1	0	0	0	0	0	0	1	1	0	0	0	0	0	[39]	132
**3**	**Bourrouilla**	b3	0	1	0	1	0	0	0	0	0	0	1	0	0	0	0	0	0	[39]	82
**3**	**Bourrouilla**	c	1	1	0	1	0	0	0	0	0	0	1	0	0	0	0	0	0	[39]	104
**4**	**Isturitz**	Late Magdalenian	1	1	0	0	0	0	0	0	0	0	1	0	0	0	0	0	0	[28]	8
**4**	**Isturitz**	Middle Magdalenian	1	1	0	1	0	0	0	0	0	0	1	0	1	0	0	0	0	[28]	7
**5**	**Espélugues**	-	0	1	0	0	0	0	0	0	0	1	0	1	1	0	0	0	0	[40]	-
**6**	**Bois du Cantet**	boyau 7	1	1	0	1	0	0	1	0	0	0	0	0	1	1	1	0	0	[41]	160
**6**	**Bois du Cantet**	Magdalenian level	0	1	0	0	0	0	1	0	0	0	1	0	1	0	0	0	0	[41]	-
**7**	**La Vache**	"Salle Monique"	0	1	0	0	0	0	0	1	0	0	1	0	1	1	0	0	0	[42]	-
**8**	**Conques**	C2	0	1	0	0	0	0	0	1	0	1	0	1	1	1	0	0	0	[43]	-
**8**	**Conques**	C3	0	1	0	0	0	0	0	1	0	1	1	1	1	1	0	0	0	[43]	-
**9**	**Abeurador**	5 central area	1	1	0	1	1	0	1	0	0	1	1	0	1	1	1	0	0	[31]	135
**9**	**Abeurador**	7 central area	1	1	0	0	1	0	0	0	0	1	1	0	1	1	1	0	0	[31]	127
**9**	**Abeurador**	8 central area	1	1	0	1	1	0	1	0	0	1	1	0	1	1	1	0	0	[31]	459
**9**	**Abeurador**	10 central area	1	1	1	1	0	0	0	0	1	1	1	0	1	1	1	0	0	[31]	550
**10**	**Grotte du Salpêtre de Pompignan**	1A	0	1	1	1	1	0	1	0	0	1	1	0	1	1	1	0	0	[28]	193
**11**	**Abri Fontalès**	-	0	1	1	0	0	0	0	0	1	1	1	0	1	0	0	0	0	[44]	-
**12**	**Peyrugues**	14	1	1	0	0	0	0	0	0	1	1	1	1	0	1	0	0	0	[45]	-
**12**	**Peyrugues**	12	1	1	0	0	0	0	0	0	0	0	1	1	0	1	0	0	0	[45]	-
**12**	**Peyrugues**	16	0	1	0	0	0	0	0	0	1	1	1	0	0	0	0	0	0	[45]	-
**12**	**Peyrugues**	18	1	1	0	0	0	0	0	0	0	1	1	0	0	1	0	0	0	[45]	-
**12**	**Peyrugues**	20	0	1	0	0	0	0	0	0	0	1	1	1	0	1	0	0	0	[45]	-
**12**	**Peyrugues**	22	0	1	0	0	0	0	1	1	1	1	1	0	0	0	0	0	0	[45]	-
**12**	**Peyrugues**	3	1	1	0	0	0	0	0	0	0	1	1	0	1	1	0	0	0	[45]	-
**12**	**Peyrugues**	5	1	1	0	0	0	0	0	0	0	1	1	1	0	0	0	0	0	[45]	-
**12**	**Peyrugues**	6	1	1	0	0	0	0	0	0	0	0	1	0	0	0	0	0	0	[45]	-
**12**	**Peyrugues**	9	1	1	0	0	0	0	0	0	0	1	1	1	0	1	0	0	0	[45]	-
**13**	**Peyrazet**	C2	1	1	0	0	0	0	1	0	0	0	1	0	1	0	0	1	0	Unpublished	[100–150]
**13**	**Peyrazet**	C3	0	1	0	0	0	0	1	0	0	0	1	0	0	1	0	0	0	Unpublished	[100–150]
**13**	**Peyrazet**	C4sommet	1	1	0	0	0	0	1	0	0	0	1	0	1	1	0	0	1	Unpublished	[200–250]
**13**	**Peyrazet**	us 7	1	1	0	0	0	0	1	0	0	1	1	0	0	0	0	0	1	Unpublished	[200–250]
**14**	**Roc de Combe**	C2	0	1	1	0	0	0	0	0	0	0	0	0	1	0	1	0	0	[29,46]	7
**14**	**Roc de Combe**	C5	1	1	1	0	0	0	0	0	0	0	1	0	0	0	0	0	0	[29,46]	54
**14**	**Roc de Combe**	C7	1	1	1	0	0	0	0	0	0	1	1	0	0	1	0	0	0	[29,46]	294
**14**	**Roc de Combe**	C8	0	1	1	0	0	0	0	1	0	1	1	0	1	1	0	0	0	[29,46]	31
**15**	**Le piage**	F	0	1	1	0	0	0	0	0	0	0	1	0	0	0	0	0	0	[47]	11
**16**	**Combe-Grenal**	C20	1	1	0	0	0	0	0	0	0	1	1	0	0	0	0	0	0	[29,46]	20
**17**	**Les Fieux**	F1C	1	1	0	0	0	0	0	0	1	0	1	1	0	0	0	0	0	Unpublished	-
**18**	**Borie del Rey**	Laborian	0	1	0	0	0	0	0	0	0	1	1	0	0	0	0	0	0	[48]	-
**19**	**Le Flageolet 1**	I-III	1	1	1	0	0	0	0	0	0	0	1	0	1	0	0	0	0	[29,46]	29
**19**	**Le Flageolet 1**	IV	1	1	1	0	0	0	0	0	0	0	1	0	1	0	0	0	0	[29,46]	22
**19**	**Le Flageolet 1**	V	0	1	1	0	0	0	0	0	0	0	1	0	0	0	0	0	0	[29,46]	13
**19**	**Le Flageolet 1**	VII	0	1	1	0	0	0	0	0	1	0	1	0	0	0	0	0	0	[29,46]	6
**19**	**Le Flageolet 1**	VIII-IX	1	1	1	0	0	0	0	1	0	1	1	0	1	1	0	0	0	[29,46]	290
**19**	**Le Flageolet 1**	XI	1	1	1	0	0	0	0	1	0	1	1	0	0	1	0	0	0	[29,46]	30
**19**	**Le Flageolet 2**	II	0	1	0	0	0	0	0	0	0	0	1	0	1	0	0	0	0	[29,46]	5
**19**	**Le Flageolet 2**	IX	1	1	1	1	0	0	0	0	0	1	1	0	0	1	1	0	0	[29,46]	27
**20**	**Moulin du Roc**	"Couche Bigarrée"	1	1	0	0	0	0	0	0	0	0	1	0	1	0	1	0	0	[49]	64
**20**	**Moulin du Roc**	"Couche Brune S.I."	1	1	1	1	0	0	1	0	0	0	1	1	1	1	1	0	0	[49]	119
**20**	**Moulin du Roc**	"Couche Jaune"	1	1	1	0	0	0	0	0	0	0	1	0	0	0	0	0	0	[49]	42
**21**	**La Ferrassie**	D	1	1	1	0	0	0	0	0	0	1	1	0	0	0	0	0	0	[29,46]	8
**22**	**Abri Pataud**	3 (perigordian VI)	0	1	0	0	0	0	0	0	0	0	1	0	0	1	0	0	0	[50]	-
**22**	**Abri Pataud**	5 (perigordian IV)	0	1	0	0	0	0	0	0	0	0	1	0	0	0	0	0	0	[50]	-
**22**	**Abri Pataud**	7/8	0	1	1	1	0	0	0	0	0	1	1	0	1	1	0	0	0	[50]	-
**23**	**Grotte de Comarque**	-	1	1	0	0	0	0	0	0	1	0	1	0	0	1	0	0	0	[51]	-
**24**	**Abri du Facteur**	Aurignacian	0	1	0	0	0	0	0	0	0	1	1	0	0	1	0	0	0	[52]	-
**24**	**Abri du Facteur**	Perigordian V	0	1	0	0	0	0	1	0	0	1	1	0	0	0	0	0	0	[52]	-
**25**	**Castanet**	Aurignacian A/Aurignacian 1	0	1	0	0	0	0	0	1	0	0	1	0	0	0	0	0	0	[26]	-
**26**	**Combe-Saunière I**	III	1	1	1	0	0	0	0	1	0	1	1	0	1	1	0	0	0	[29,46]	54
**26**	**Combe-Saunière I**	IV	1	1	1	0	0	0	0	1	1	1	1	0	0	1	0	0	0	[29,46]	291
**26**	**Combe-Saunière I**	VA	0	1	1	0	0	0	0	0	1	0	1	0	0	0	0	0	0	[29,46]	13
**27**	**Pont d'Ambon**	2-3A	1	1	1	0	0	0	1	1	0	0	1	0	1	1	0	0	0	[29,46]	595
**27**	**Pont d'Ambon**	3BC	1	1	1	0	0	0	1	0	0	0	1	0	1	1	0	0	0	[29,46]	79
**27**	**Pont d'Ambon**	4	1	1	1	0	0	0	0	1	0	0	1	0	1	1	0	0	0	[29,46]	45
**28**	**Roc de Marcamps**	2	1	1	1	0	0	0	0	1	0	0	1	0	1	1	0	0	0	[29,46]	206
**28**	**Roc de Marcamps**	3	1	1	1	0	0	0	0	1	0	0	1	0	0	0	0	0	0	[29,46]	498
**28**	**Roc de Marcamps**	4	1	1	1	0	0	0	0	1	0	0	1	0	1	1	0	0	0	[29,46]	358
**28**	**Roc de Marcamps**	5	1	1	1	0	0	0	0	1	0	0	1	0	0	0	0	0	0	[29,46]	114
**28**	**Roc de Marcamps**	7–8	0	1	1	0	0	0	0	0	1	0	1	0	0	0	0	0	0	[29,46]	44
**29**	**La Roche à Pierrot**	EJJ	0	1	1	0	0	0	0	0	0	0	1	0	0	0	0	0	0	[29,46]	109
**29**	**La Roche à Pierrot**	EJOP	0	1	1	0	0	0	0	0	0	0	1	0	0	0	0	0	0	[29,46]	68
**30**	**Abri Pintaud-Gaudry**	2	1	1	1	1	0	0	1	1	1	0	1	0	1	1	0	0	0	[29,46]	281
**31**	**Abri Ragout**	B	1	1	1	0	0	0	0	1	0	0	1	0	0	0	0	0	0	[53]	23
**32**	**Bois Ragot**	5	1	1	1	1	0	0	1	1	1	0	1	0	1	1	0	0	0	[54]	119
**33**	**Taillis des Coteaux**	IIG	1	1	1	0	0	0	1	1	1	1	1	0	0	1	0	1	0	[55,56]	823
**33**	**Taillis des Coteaux**	IIIA	1	1	1	0	0	0	1	1	1	1	1	1	1	1	0	0	0	[55,56]	2170
**33**	**Taillis des Coteaux**	IIIB	1	1	1	0	0	1	0	1	1	0	1	1	0	0	0	0	0	[55,56]	979
**33**	**Taillis des Coteaux**	Vd	1	1	1	0	0	0	0	1	1	0	1	0	0	0	0	0	0	[55,56]	31
**33**	**Taillis des Coteaux**	VIA	1	1	1	0	0	0	0	1	1	0	1	0	0	0	0	0	0	([55,56]	34
**33**	**Taillis des Coteaux**	VIG	1	1	1	0	0	0	0	1	1	1	1	0	0	0	0	0	0	[55,56]	188
**33**	**Taillis des Coteaux**	VIIA	1	1	1	0	0	0	0	1	1	0	1	0	1	1	0	0	0	[55,56]	138
**34**	**Abri Garenne (Grand Abri)**	B1/B2	1	1	1	0	0	0	0	1	1	1	1	0	0	0	0	0	0	[57]	-
**35**	**Les Cottés**	US 2	0	1	1	0	0	0	0	0	1	0	0	0	0	0	0	0	0	Unpublished	10
**35**	**Les Cottés**	US 4 upper phase	0	1	1	0	0	0	0	1	0	0	1	0	1	0	0	0	0	Unpublished	28
**35**	**Les Cottés**	US 4 lower phase	0	1	1	0	0	0	0	1	0	0	1	0	1	0	0	0	0	Unpublished	10
**35**	**Les Cottés**	US 6	0	1	1	0	0	0	0	1	0	0	1	0	0	0	0	0	0	Unpublished	14
**36**	**Abri Fritsch**	-	1	1	1	0	0	0	0	0	1	0	0	0	0	0	0	0	0	[28]	-

### Database and faunal list

Southwestern France is currently structured by a diversity of landforms, stream networks, and microclimates responsible for local variations in extant small mammal communities. On a regional scale, the rodent community is relatively homogeneous. The main differences are the limited presence of the pine vole (*Microtus (Terricola) subterraneus*) in the northern part of the region, whereas the Mediterranean pine vole (*Microtus (Terricola) duodecimcostatus*) is restricted to the southeastern part as is the snow vole (*Chionomys nivalis*), a rupicolous species which inhabits middle and high latitudes of the Pyrenees Mountains [37].

For the present study, a database of 94 stratigraphic units was compiled from 37 archaeological and paleontological sites (Table 1) located in southwestern France (Fig 1). All the sites are located at altitudes lower than 250 meters, except for four sites (La Vache, altitude = 580 m; Espélugues, altitude = 430 m; Bois du Cantet, altitude = 380 m; Salpêtre de Pompignan, altitude = 380 m). Only assemblages with at least one radiocarbon date have been considered. At least twenty different rodent species belonging to five families inhabited southwestern France during the Late Pleistocene. Today, seven of them do not inhabit southwestern France: the northern birch mouse (*Sicista betulina*), the common hamster (*Cricetus cricetus*), the striped field mouse (*Apodemus agrarius*), the ground squirrel (*Spermophilus* sp.), the root vole (*Microtus oeconomus)*, the narrow-headed vole *(Microtus gregalis)* and the collared lemming *(Dicrostonyx torquatus)*. The last four species, which today inhabit significantly higher latitudes than southwestern France, are considered in this paper to be cold-climate rodents. On the contrary rodents still living in southwestern France, with a distribution extending up to 60°N of latitude, including the fat dormouse (*Glis glis)*, the garden dormouse *(Eliomys quercinus*), the Mediterranean pine vole *(M*. *(T*.*) duodecimcostatus)*, the pine vole (*M*. *(T*.*) subterraneus*), the water vole (*Arvicola amphibius)*, the southern water vole (*Arvicola sapidus*), the bank vole (*Clethrionomys glareolus*) and the wood mouse *(Apodemus sylvaticus)* are considered as species of temperate climate.

Presence vs. absence data were preferred (Table 1), as in many studies there is no mention of the minimum number of individuals and how it could result from ecological and taphonomic processes such as predator action, seasonality, or mode of burial [37,58,59]. Binary codes 0 and 1 were consequently assigned respectively to the absence and presence of a given rodent species within a stratigraphic unit. Following the principle of “total evidence”, all species were included in the database for the multivariate analyses, except *Apodemus agrarius*, which was only identified at Bouziès-Q in the Quercy region without full detail on the associated small mammals [60]. *Microtus agrestis* and *M*. *arvalis* occurrences were computed as *M*. *arvalis/agrestis* due to the lack of differentiation between these taxa in many studies.

The database used was built using the maximum number of archaeological stratigraphic assemblages that are dated by radiocarbon methods, and thus includes archaeological sites where taphonomic information is lacking. Even if different reasons might explain the absence of a rodent species in a faunal list (other than its total absence), such as sampling biases or preferential hunting strategies of their predators (e.g. weasel), we argue that the statistical analysis of such a large dataset should reduce the potential interpretive biases regarding observed changes in the community structures of rodents.

### Building a time scale with increasing time-intervals

In the present study, changes in faunal communities are investigated on a regional scale by combining the datasets from several archaeological and palaeontological sites into time periods, in order to obtain a representative faunal spectrum of a region for a specific period [61].

In most palaeoecological works, stratigraphic units are either grouped by predetermined periods (such as the Last Glacial Maximum or the Bølling), by using limits defined by other climatic proxies (such as oxygen isotope compositions of ice cores; i.e. [62]) or with user-chosen specific time-intervals (e.g., time intervals of 500, 1000, or 5000 years). Nevertheless, these two methods have several disadvantages. The predetermined periods are often defined by proxies of global changes that are not necessarily relevant to regional-scale studies where environments are influenced by a combination of climatic, geographic, topographic, and geologic factors. On the other hand, the use of preset time-intervals is complicated by the varying resolution of radiocarbon dates through time (e.g. radiocarbon dates for older periods are characterized by larger standard errors). Consequently, the size of the intervals is often too wide for younger periods, so that some important information might be lost and subtle changes unseen, while on the contrary, datasets for older periods can be over-interpreted as the temporal resolution is too wide relative to the resolution of the raw data.

To overcome these issues, this study relies on a time-scale that is based on time-intervals that are independent of predetermined periods. These time-intervals have varying bin sizes according to time to adapt to the differential chronological resolution of the dataset. The temporal resolution (e.g. the bin width) was set per time period according to the resolution of the radiocarbon dates. A relationship exists between the age of a radiocarbon date and the standard error of this date, as older radiocarbon dates tend to be associated with larger standard errors. Here, we exploit this relationship to propose a time-scale divided into a succession of bins (called here time-intervals) of decreasing width progressively through time from 50 to 10 ky cal. BP.

To calculate these time-intervals, three successive steps were taken:

1. A linear regression (reduced major axis) was calculated between the available radiocarbon dates determined from the studied stratigraphic units (S1 Table) and the square root of the standard error of these dates (S1 Fig):
  $$y=0.00129x-6.28240;R^{2}=0.624;n=145;p=3.5\times 10^{-32}$$
  Here y is the square root of the standard error of date and x is the radiocarbon date. Radiocarbon dates from Les Cottés (Vienne) have recently been obtained [63], and their standard errors are significantly smaller compared to others compiled in the database. They have therefore been excluded from the database when calculating the regression equation (Eq 1).
2. The starting point was set at 10,000 ± 40 yr BP.
3. The limits of the time-intervals were computed using Eq 1 and the starting point. Each time-interval is defined by a starting point (e.g. 10,000) and an end point that is equal to the sum of the starting point itself and two times the typical standard error for radiocarbon dates at this starting point, as given by the regression formula (e.g. the standard error for 10,000 is 40, hence the end point is 10,000 + 40 x 2, so that the first interval is 10,000–10,080).

Between 50 and 10 kyr, a total of 56 time-intervals has been calculated, with bin width decreasing progressively from 6582 to 80 years (S2 Table). A stratigraphic unit crosses over one or several time-intervals according to its duration, which is defined by the minimal and maximal radiocarbon ages obtained in each assemblage (S1 Table). All time-intervals are reported along with the total number of stratigraphic units that cross over each of them in S2 Table. For each stratigraphic unit, all chronological information was taken in consideration (radiocarbon dates, cultural attributions) in order to select minimum and maximum radiocarbon dates representative of the occupation. Radiocarbon dates identified as outliers were excluded during this process (S1 Fig). In order to be conservative, these minimal and maximal values correspond respectively to the 2.5% and 97.5% boundaries of the minimal and maximal calibrated age distributions (S1 Table). Dates have been calibrated at 2σ (95.4%) using OxCal 4.2 and IntCal13 calibration curve [64].

### Estimating the relative frequency of taxa and multivariate analyses

**Fig 2:**
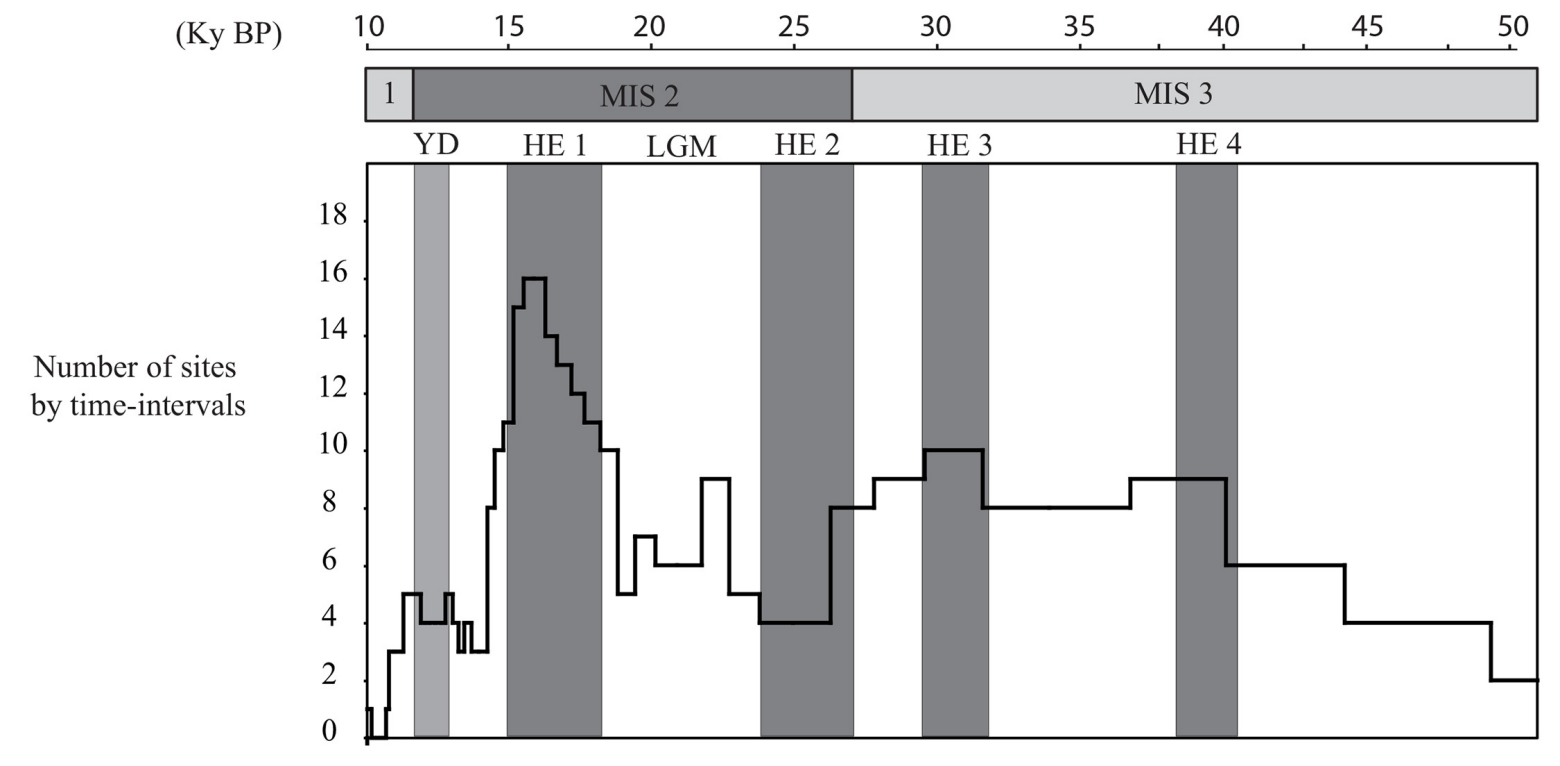
Temporal variations of the number of archeological sites that cross over each time interval. Abbreviations are: MIS = Marine Isotope Stage; YD = Younger Dryas; HE = Heinrich event; LGM = Last Glacial Maximum. Grey intervals indicate the four Heinrich events and the Younger Dryas.

**Fig 3:**
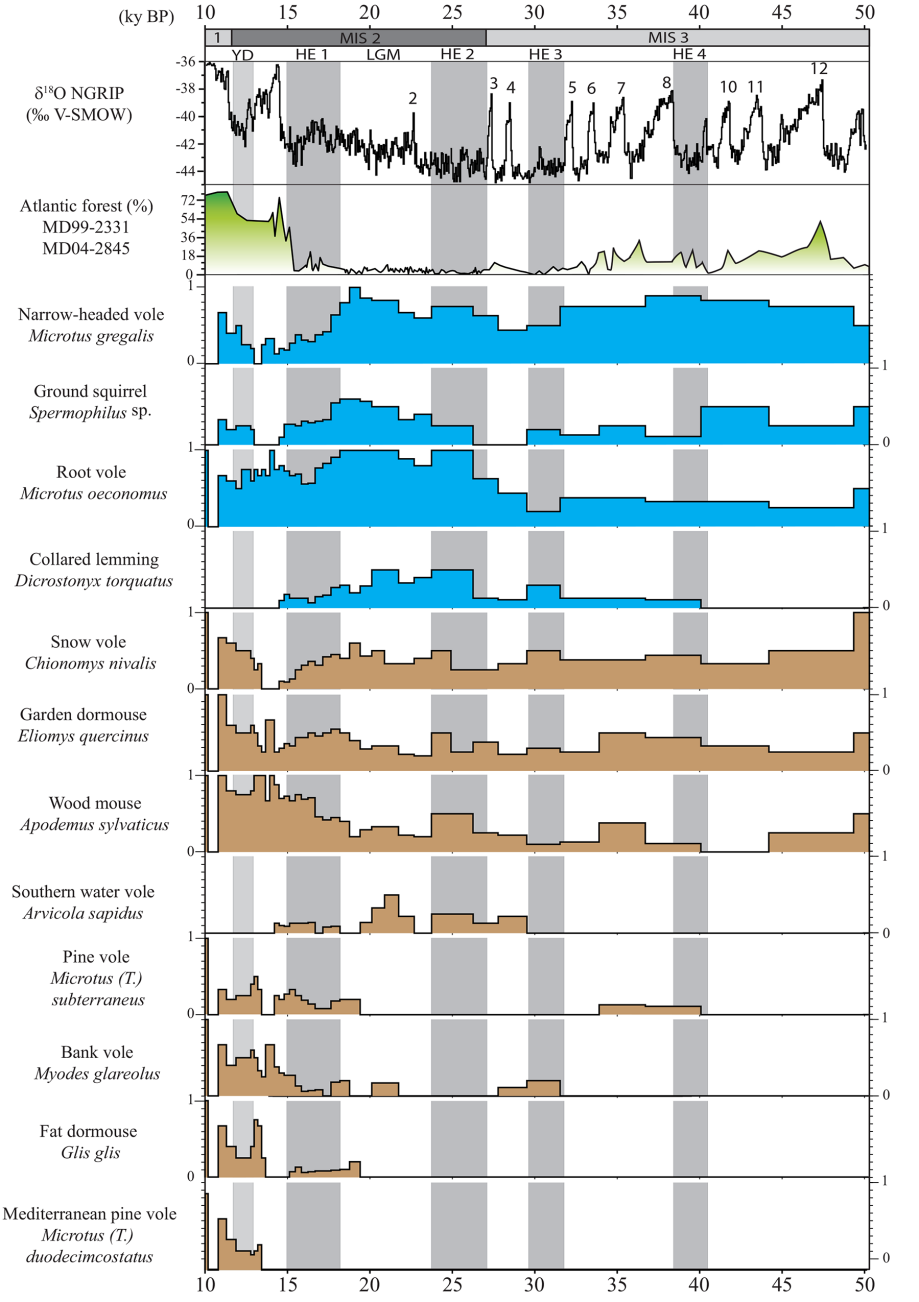
Temporal variations of the occurrences of rodent species in southwestern France during the Late Pleistocene, illustrated by the computed relative frequency of taxa (scale 0–1, cf. text for details). For comparison and discussion are also reported the oxygen isotope compositions of the Greenland ice cores (NGRIP) and the reconstructed Atlantic forest cover based on pollen assemblages from deep-sea cores MD04-2845 and MD99-2331 [6]. Numbers 2–12 refer to the Dansgaard-Oeschger interstadials. Rodent species still present nowadays in the study region are represented in brown, whereas the rodent species that became extinct are represented in blue. Grey intervals indicate the four Heinrich events and the Younger Dryas event. The hatched area indicates time-intervals with no data. The dashed black line represents the separation between Bølling and Allerød periods. See Fig 2 for abbreviations.

One might assume that the more abundant a given rodent species is in a region, the higher its probability of capture by predators in several of the region’s sites. Consequently, the proportion of sites in which a given rodent is found could be interpreted as signal strength for that species, assuming that sites are equivalent. For a given rodent species, its low frequency in the region indicates a limited distribution either in few locations or in small populations, whereas on the contrary, a high frequency indicates a large distribution and favorable environmental conditions for this species. We thus use the relative frequency of taxa, calculated for each time-interval and each species as follows: the number of stratigraphic units assigned to the time-interval considered in which the species is present divided by the total number of stratigraphic units for the time-interval considered (Fig 2). Consequently, the presence of a species is reduced to the interval [0; 1], corresponding respectively to its absence or presence in all the studied stratigraphic units from one time-interval, with a gradient of abundance between the two extreme values 0 and 1 (Fig 3). In order to be accurate, we calculated 95% confidence intervals for the proportion of each species from each time-interval (S2 Fig), as a function of sample size (here, the number of stratigraphic units) and the number of items in the sample, using the statistical table published in Sokal and Rohlf [65]. For comparison with the temporal variation of relative frequency of taxa presented in Fig 3, we also reported the oxygen isotope compositions of the Greenland ice cores (NGRIP) and the reconstructed Atlantic forest cover based on pollen assemblages from deep-sea cores MD04-2845 and MD99-2331 [6].

The variation in small mammal communities has been investigated by combining two multivariate approaches, both of which are based on the number of stratigraphic units in which each species is present for each time-interval (S3 Table). The first one is a factor analysis exploring the faunal changes that influence communities without any temporal constraint, whereas the second approach is a temporally constrained cluster analysis computed using the Bray-Curtis dissimilarity index in order to hierarchically cluster the time-intervals. These statistics were computed using Past software (Past v.3.05, [66]).

It is worth noting that the estimation of the frequencies of a given taxa can be influenced by the number of stratigraphic units used in the calculation. Thus, for both multivariate analyses, time-intervals constituted by fewer than three stratigraphic units have been excluded. As noted, an individual stratigraphic unit may be included in several time-intervals, but we do not consider the statistical independence of data as a major issue, as we used AFC and cluster analyses solely as methods for exploring our dataset. Besides, the purpose here is not to estimate precisely the presence proportion of particular rodent species, but to reconstruct the main changes in their proportions through time. As a consequence, only the high magnitude changes have been considered in this study.

## Results

### Evolutionary patterns in the abundance of rodent species

Twelve rodent species, exhibiting noticeable past relative frequency variations, were analyzed for temporal variation of their occurrence in southwestern France (Fig 3). In detail, it is observed that each species, whether still extant or extinct (S2 Fig), is characterized by a distinct temporal pattern of distribution that can be categorized in three main groups.

The first group includes *Glis glis*, *Clethrionomys glareolus*, *Microtus (Terricola) subterraneus*, and *Microtus (Terricola) duodecimcostatus* that were absent during a large part of the Late Pleistocene. These four species appeared progressively after the Last Glacial Maximum (LGM). For example, *C*. *glareolus* extended its distribution at the end of the Heinrich event 1 and throughout the Bølling-Allerød interval, while *M*. *(T*.*) duodecimcostatus* became apparently more widespread from the beginning of the Holocene. Few occurrences of *C*. *glareolus* and *M*. *(T*.*) subterraneus* are observed during MIS 3, probably related to milder periods or events. The pattern presented by *Arvicola sapidus* is difficult to explain so far, with its unexpected increase in abundance during the LGM, while today this rodent only occurs in the freshwater habitats of Portugal, Spain and France.

By contrast, the second group is notably constituted by *Eliomys quercinus* and *Apodemus sylvaticus* that were widespread during the Late Pleistocene, but most often occurred in low abundances, even though three peaks of increasing abundance were recorded at 50 ky BP, 34 ky BP and around 25 ky BP (Fig 3). These two rodent species became abundant at the end of the LGM. *Chionomys nivalis* is characterized by a different pattern of distribution, close to other species that disappeared during the Holocene. This rodent is indeed still present in southwestern France; however it is mainly geographically restricted to locations with large seasonal amplitudes of temperature and important exposures to sunlight, such as the elevated areas of the Pyrenean Mountains.

The last group is composed by rodent species, which were all abundant during the LGM, and recently disappeared from southwestern France (Fig 3). The occurrence of *Microtus oeconomus* can be divided into three distinct phases with: first, a scarce presence until the Heinrich event 2; second, a common occurrence until the beginning of the Holocene when it disappeared. *Spermophilus* sp. was always present during the Pleistocene, during the Marine Isotope Stage 3 (MIS 3) and the LGM. The presence of *Microtus gregalis* is prominent until the Heinrich event 1 during which its abundance began to decrease until the Younger-Dryas event, which was marked by a sharp peak in its abundance, then followed by a rapid decline and its disappearance during the Holocene. *Dicrostonyx torquatus* presents a unique pattern of distribution with a quasi absence in the sedimentary record older than 35 ky BP. Even if remains from this species were retrieved from archaeological sites dated to the beginning of MIS 3 [35] and MIS 4 [34], its presence was not documented between 50 and 38 ky BP. Populations belonging to this northern holarctic rodent species have been most likely strongly affected by warm climate peaks related to the Dansgaard-Oeschger events of MIS 3 [67], which apparently contributed to their extinction in southwestern France. The abundance of *D*. *torquatus* increased during Heinrich events 3 and 2, then decreased at the end of the LGM. This rodent finally disappeared during the Bølling event (Fig 3).

### Structural changes of rodent communities

**Fig 4:**
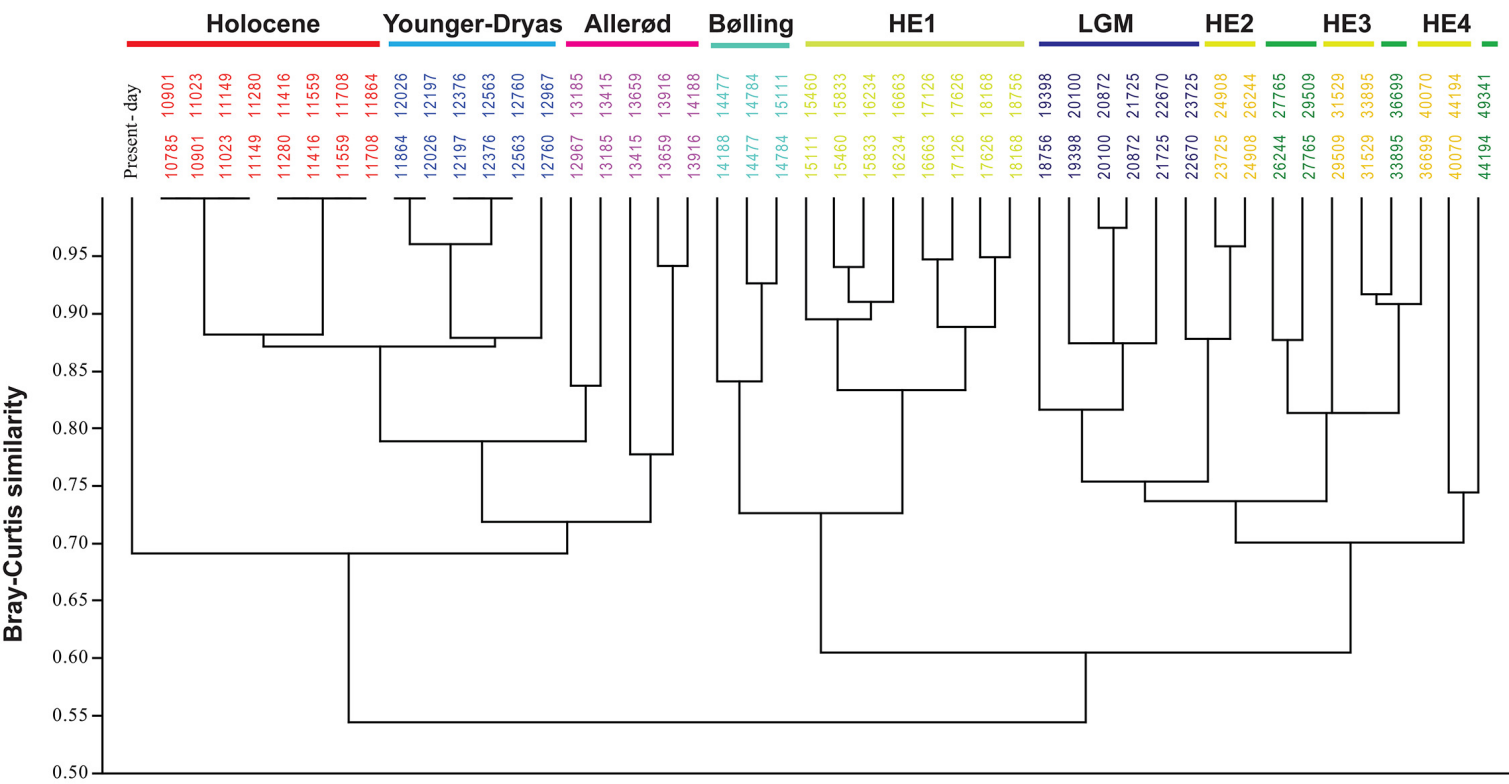
Cluster analysis based on Bray–Curtis dissimilarity and constrained by the succession of time-intervals. Numbers correspond to the time-intervals. The different periods are distinguished by the colors used in Fig 5. The three green periods that are not named in the figure correspond to periods between Heinrich events 5 and 4, between Heinrich events 4 and 3, Heinrich events 3 and 2. See Fig 2 for abbreviations.

By using cluster analysis, two important changes of communities (Fig 4) have been recognized at about 18–19 ky BP and 14.1 ky BP. They correspond to known periods of climatic changes in the northern hemisphere. Three communities have been identified. The first community occurred in a time span between 50 and 19 ky BP, and is characterized by high proportions of rodent species from cold climates (*D*. *torquatus*, *M*. *gregalis*, *M*. *oeconomus* and *Spermophilus* sp.). Results from cluster analysis show no important statistical difference between the LGM and the period between 50 and 18 ky BP. The first shift corresponds to the establishment of a second community defined by a recurrent presence of temperate climate species (C. *glareolus*, *M*. *(T*.*) subterraneus*, *A*. *sylvaticus* and *E*. *quercinus*) during both the Heinrich event 1 and Bølling events. Finally, after the second shift at 14.1 ky BP, the Allerød and the Holocene are primarily marked by the presence of temperate species such as *G*. *glis*, and *M*. *(T*.*) duodecimcostatus*.

**Fig 5:**
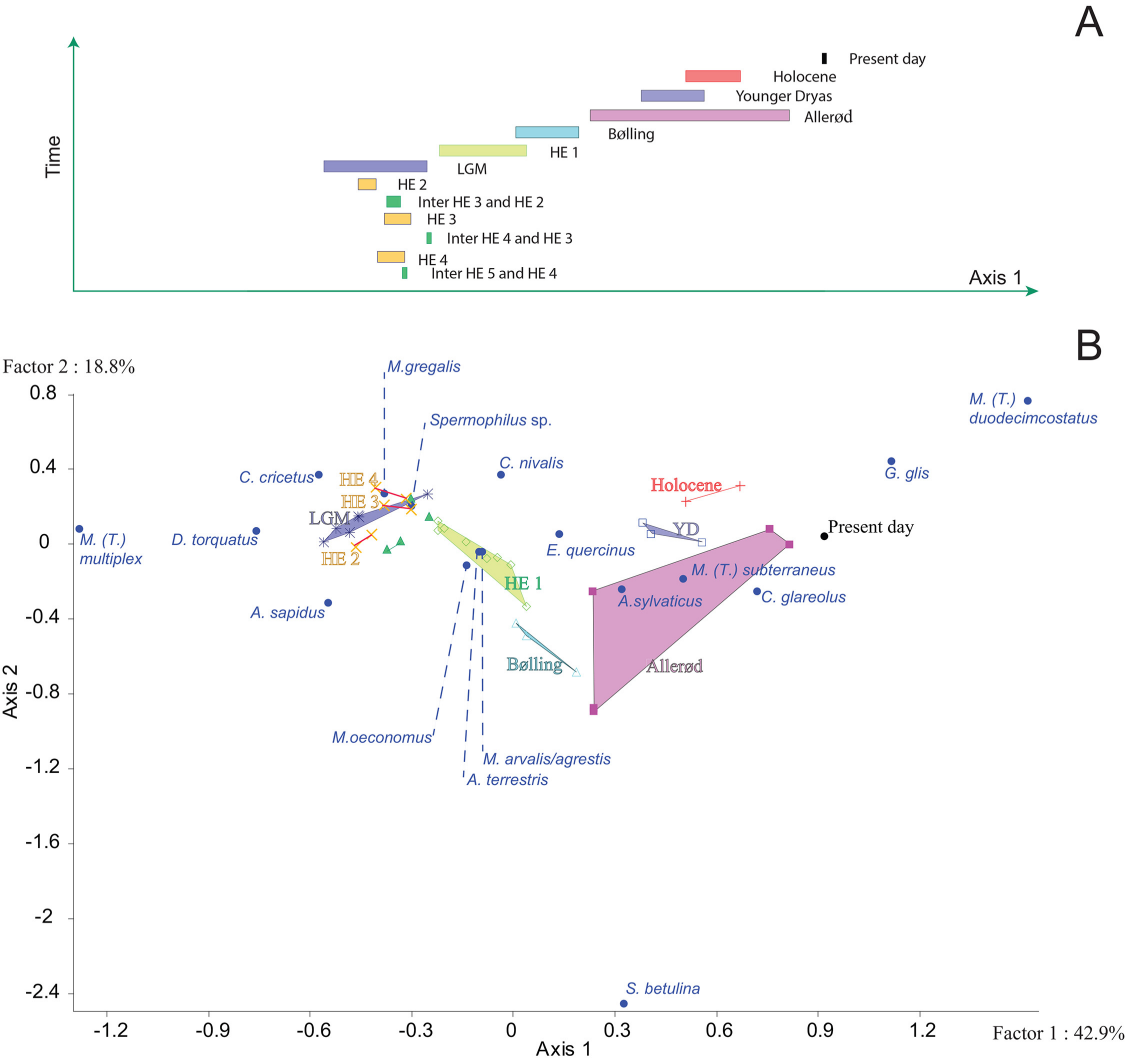
Correspondence analysis based on rodent associations defined for the species (circles) and each time-interval, gathered by following periods. (▲): periods between Heinrich events 5 and 4; (x): Heinrich events 4 (40.2–38.3 ky BP); (▲): periods between Heinrich events 4 and 3 (38.3–32.7 ky BP); (x): Heinrich events 3 (32.7–29.0 ky BP); (▲): periods between Heinrich events 3 and 2 (29.0–26.0 ky BP); (x): Heinrich events 2 (26.0–24.0 ky BP); (_*_): Last Glacial Maximum (24.0–18.0 ky BP); (◊): Heinrich event 1 (18.0–14.7 ky BP); (Δ): Bølling (14.7–13.7 ky BP); (■): Allerød (13.7–12.9 ky BP); (□): Younger-Dryas (12.9–11.7 ky BP); (+): Holocene (11.7–10.9 ky BP). Time-intervals constituted by less than three stratigraphic units were excluded from the statistical analysis. See Fig 2 for abbreviations. A) Succession of periods obtained by time-intervals reported against Axis 1. B) Axis 1 reported against the Axis 2.

The variation in small mammal communities has been investigated, as a second step, by using a factor analysis based on communities from each time-interval (Fig 5). The first and second axes explain more than 70% of the variance. The first axis is based on the opposition between *M*. *(T*.*) multiplex*, *C*. *cricetus* and some cold-adapted species (such as *M*. *gregalis* and *D*. *torquatus)* on one hande and those of temperate climatic conditions (such as *G*. *glis* and *M*. *(T*.*) duodecimcostatus*) on the other hand. This first axis separates a group composed of the Holocene, Younger-Dryas and Allerød rodent assemblages from a second one composed of the periods dated between 50 ky BP and the Bølling. This axis shows equally that the intermediate group identified in cluster analysis, which extends from 18 to 14.1 ky BP, is located at the center of the factor analysis, which is characterized by the association of both cold-climate species (such as *M*. *oeconomus*) and temperate-climate species (*M*. *arvalis/agrestis* or *E*. *quercinus*). The second axis opposes *C*. *nivalis*, *G*. *glis* and *M*. *(T*.*) duodecimcostatus* from *A*. *sapidus*, and leads to a distinction of the Heinrich event 4 and Holocene from the Bølling and Allerød periods. Pleistocene events are relatively well separated even though some events show a large variance such as the LGM, even if no statistical differentiation has been observed with clustering analysis.

When patterns identified in the presence proportions of rodent species and the rodent community structure are summed up, three periods can be distinguished: a first period between 50 ky and the LGM, the period from the HE 1 to the Bølling, and the Allerød-Holocene (Fig 4). The period dated between 50 ky and the LGM is characterized by high proportions of cold climate rodent species, such as *M*. *gregalis*, *Spermophilus* sp. and *D*. *torquatus*, whereas species of temperate climate (*G*. *glis*, *M*. *(T*.*) duodecimcostatus*, *A*. *sylvaticus* and *E*. *quercinus*) are abundant during the Holocene. The period extending from the HE 1 to the Bølling shows an intermediate situation with the association of both cold and temperate climate rodent species.

### Spatial variability in rodent distribution

**Fig 6:**
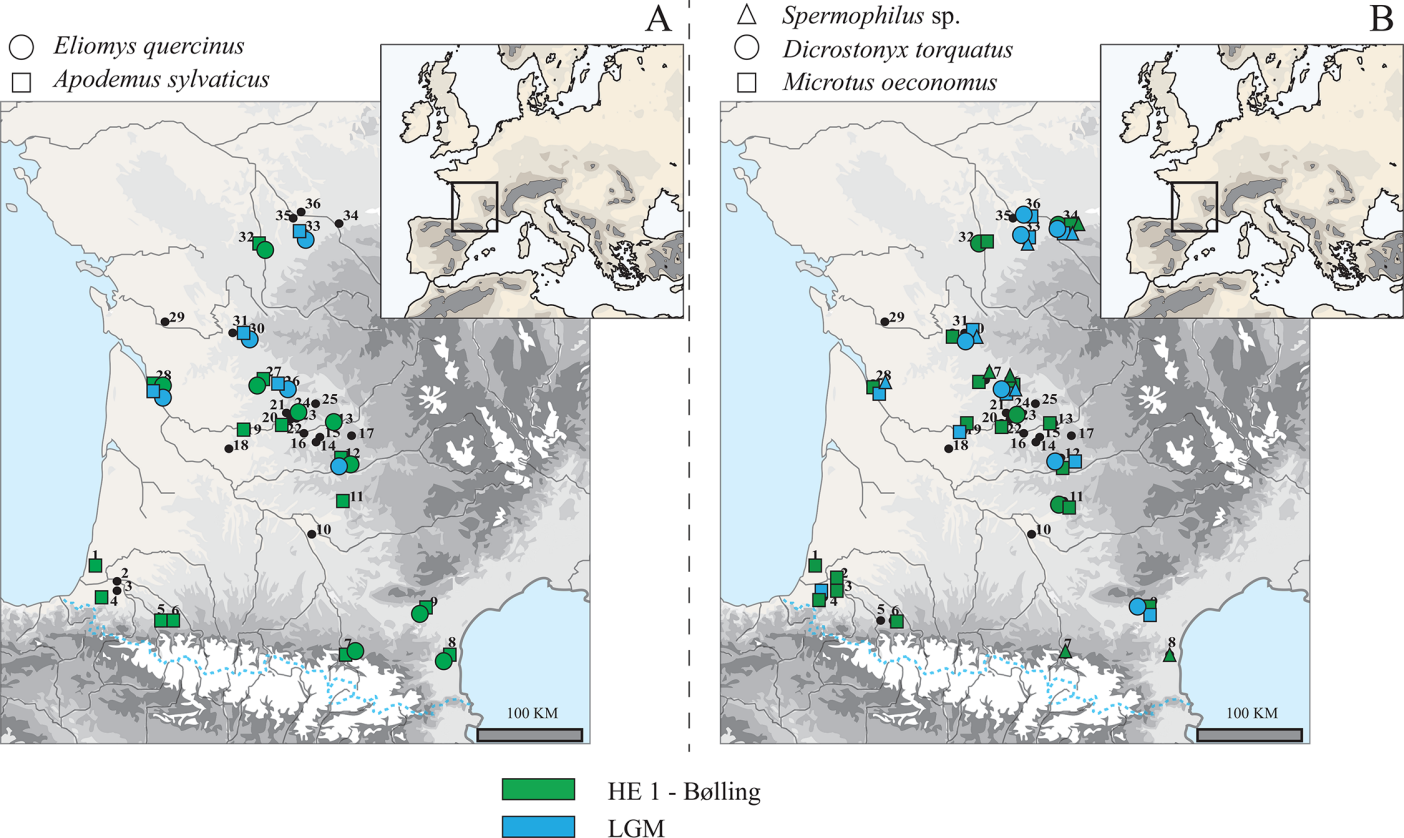
Geographic maps of the occurrences of rodent species in southwestern France during the Last Glacial Maximum (LGM) (in blue) and during the period of Bølling and Heinrich Event 1 (HE1) (in green). A) Occurrences of garden dormouse (*Eliomys quercinus*) and wood mouse (*Apodemus sylvaticus*), temperate-climate species. B) Occurrences of ground squirrel (*Spermophilus* sp.), collared lemming (*Dicrostonyx torquatus*) and root vole (*Microtus oeconomus*), cold-climate species.

At the geographic scale of southwestern France, spatial variability in the distribution of temperate versus cold-tolerant rodent species already existed during the Pleistocene. Pyrenean sites that yield Pleistocene fossils of cold-adapted small mammal species are rare and are mainly located on the eastern side of the mountain range, with occurrences of *D*. *torquatus* and *M*. *gregalis* in level 10 of l'Abeurador [31] (site 9, Fig 6). *Spermophilus* sp. was identified in la Vache and Conques [42,43] (sites 7 & 8, Fig 6), but was not collected in LGM sites. This observation probably results from a problem of sampling as is presence is attested in the northeastern Iberia during the LGM [68]. In contrast, the colder-climate species retrieved in the western part of the Pyrenean Mountains are only *M*. *(T*.*) subterraneus* and *M*. *oeconomus*, of which the latter also had a geographic distribution extending to Spain during the Late Pleistocene [69,70,71]. The two temperate-climate species *A*. *sylvaticus* and *E*. *quercinus* have been recovered in stratigraphic units dated to the LGM, such as in level IIIA of Taillis-des-Coteaux (Site 33, Fig 6). These two species show a similar distribution, except in the eastern part of Pyrenean Mountains where no sites has yielded *E*. *quercinus* (Fig 6). To summarize, temperate-climate species seem to exhibit a more limited distribution during the LGM, and they then expand during the HE 1 and the Bølling. Among the cold-climate species, the distribution of *M*. *oeconomus* seems to stay rather stable, whereas *D*. *torquatus* distribution apparently contracts. The *Spermophilus* sp. distribution is still questioned and the apparent expansion observed between LGM and Bølling-HE1 may result from a lack of data in this region.

## Discussion

### Temporal changes in ecosystems

Understanding how ecosystem changes were triggered by short-term events goes far beyond any analysis performed at a single biological or ecological scale [72,73,74,75]. Cross-scale studies based on fossils constitute attractive challenges in order to understand the dynamics of past ecosystems [76,77,78,79,80,81]. Our study investigates the potential impacts on small mammals of rapid and large magnitude climatic changes that took place between 50 and 10 ky BP at the population, species, and community levels.

The occurrence of rodents varied continuously throughout the time span considered (Fig 3). The period between 50 and 19 ky BP reveals a relative stability in the structure of rodent communities, with little changes in content of faunal lists on a regional scale. It is possible that this stability might be related to the difficulty in detecting changes because of the relatively small number of sites providing small mammals for study, as well as the large dating errors obtained for the MIS 3. However, these results most likely suggest that rapid climatic events did not fundamentally modify the communities of small mammals such as rodents (Fig 5), but have nevertheless influenced the presence of some peculiar species in the region. The observed variations correspond to modifications of rodent populations at a local scale, which distributions were extended or reduced in accordance to more or less favorable local climate conditions. Studies of ancient DNA from small and large mammals have revealed, indeed, a far more complex history of faunal population dynamics than would have been predicted otherwise (e.g. [67,82,83,84,85]). Each species responded independently either directly to a given climatic event in agreement with their climatic thresholds at which tolerance levels are exceeded, or indirectly, mediated for example by interspecific interactions with predators, competitors or food sources. For example, in the specific case of *D*. *torquatus*, the study of its ancient DNA from specimens sampled in Belgium and England suggests that repeated extinctions of their populations took place during the successive Dansgaard-Oeschger warming events [67]. In addition, this study suggested that the occurrence of *D*. *torquatus* was extended to the Heinrich events 2 and 3, thus supporting the hypothesis that the arid and cold climatic conditions of these events [8,86,87], facilitated its dispersal during the Late Pleistocene [35,88,89]. It is noteworthy, however, that many species show fluctuations in their relative abundance, which are frequently correlated with climatic events. Such climatic changes may have directly influenced rodent populations or indirectly impacted them through modifications of interspecific biological interactions [75,90,91]. With regard to southwestern France, impacts of rapid climatic changes remained limited enough to preclude any complete renewal of the rodent community because these events either took place within a short time or were of too low a magnitude. The two major shifts observed in the structure of rodent communities took place between the LGM and the HE 1, and between the Bølling and the Allerød (Figs 3 and 4). The former community was mainly composed of cold rodent species while the latter was dominated by temperate species that are still living today in southwestern France. The period dated from HE 1 to Bølling presents an intermediate community constituted by the mix of extinct and extant rodents, therefore forming a rodent community with no modern analogue. It is worth noting that during this period no important break is observed during which all cold-climate species would have disappeared and been replaced by temperate species. On the contrary, the transition was gradual with a succession of faunal changes concerning both temperate and cold-adapted species. At the end of the LGM, when ice caps were retreating, temperate species such as *A*. *sylvaticus* and *E*. *quercinus* were extending, followed next by both *M*. *(T*.*) subterraneus* and *C*. *glareolus* at the Allerød when forests were expanding, and also by *G*. *glis* and *M*. *(T*.*) duodecimcostatus* during the Holocene. The collared lemming (*D*. *torquatus)* was the first cold species disappearing before the Younger-Dryas event, attesting that the post-LGM climate warming strongly affected this species [67,92]. Other cold-climate species such as *M*. *oeconomus*, *M*. *gregalis* and *Spermophilus* sp. have been perturbed by this warming event but survived the Bølling-Allerød period until the Younger-Dryas cooling event, during which they expanded again. This study supports an intermediate position between Clementsian and Gleasonian models [93,94], which notably depend on the scale of analysis. Different communities are clearly separated (Figs 4 and 5), which is in agreement with the Clementsian model, as it predicts a sustainability of the communities that move altogether in order to track favorable climate conditions. However, the biodiversity patterns emphasized in the framework of this study support as well the Gleasonian model predicting that each living organism has its own response to a climatic change in accordance with its tolerance limits. Such a mechanism results in varying rates of adaptation possibly leading to a succession of communities continuously emerging and disappearing over time, communities that can be clearly separated on a large temporal scale [94,95].

In southwestern France, modifications of communities of large mammals began as soon as the end of the LGM with the increasing occurrence of the saiga antelope (*Saiga tatarica*) during the Heinrich event 1, which was followed by its regional extinction at the beginning of the Bølling period [96,97,98]. However, the modification of such communities most likely operated in one main step, localized at the beginning of the Allerød period. This change is also characterized by the regional extinctions of bison and reindeer populations replaced by temperate species such as red deer (*Cervus elaphus*), wild boar (*Sus scrofa*), aurochs (*Bos primigenius*) and European rabbit (*Oryctolagus cuniculus*) [98,99,100,101,102]. As observed in communities of large mammals, those of small mammals seem to be subject to a noticeable change between the Bølling and Allerød periods. However, communities of small mammals also show a prior change occurring between the LGM and the HE 1. This different response of large mammals to climate changes might be explained by their migratory capacities that allow them to move in geographical areas with more favourable climatic conditions to maintain rather steady–state populations.

### Refugia and spatial variations

As mentioned above, the change in rodent communities that took place between the Pleistocene and the Holocene in southwestern France, was not a single abrupt event but a gradual one, throughout a succession of climatic events lasting at least 5,000 years. The dynamics of this faunal succession may be related to two factors: 1) the reaction time, mainly related to the preferential environmental modes of the considered rodent species, and 2) the geographical extension of the colonization phase. It has long been considered that temperate species were only in refugia, also called “macro-refugia” [103], in the three southern Peninsulas during the last glacial period and moved to higher latitudes during the postglacial stage of re-colonization (e.g. [104]). Recently, several studies suggested the possible existence during the glacial period of refugia for temperate species, called “cryptic northern refugia” [105] and “glacial microrefugia” [103], which could have been located at higher latitude in Europe than previously expected [105,106,107], such as in England [106] or in southwestern France [108,109,110]. Our study therefore proposes that many small mammals survived within two refugia in southwestern France, which are as follows:

1. Locations restricted in size that locally hosted favorable climatic conditions [106,111] such as the Pyrenean valleys. This region presents the particularity to be separated from the northern part of southwestern France by the Landes of Gascognes, which was at some periods a sandy periglacial desert [112,113], thus potentially limiting faunal migrations and shaping their geographical distributions. The network of valleys could have offered a diversity of local and favorable microclimatic conditions, along with the presence of an altitudinal gradient of the vegetation. Similar interpretations have been already inferred from genetic studies of rodent remains; insects and small mammal populations (e.g. *C*. *glareolus*) could have therefore survived in the low altitude areas of the Pyrenean valleys during the Late Pleistocene [114,115]. Moreover, studies of large mammals from different periods of the Late Pleistocene documented similar differential geographical distributions between northern parts of southwestern of France and west-east gradient along the Pyrenean Mountains [11,98,116]. Although the corpus of this study is limited for the Pyrenean region, our study leads to a similar conclusion by highlighting the presence of fewer cold species in the southern part of southwestern France (Fig 6). The important role of Pyrenean valleys is also underscored by the persistence of temperate species such as *C*. *glareolus* and most likely *M*. *(T*.*) subterraneus*, which never reached the Ibero-Lusitanian region [117,118,119].
2. Refugia forming a mosaic of sparse stands of small populations themselves scattered in multiple regions that have climatic conditions mostly unfavorable but not lethal for the considered species. This second hypothesis has been proposed for trees [120,121] as well as for small temperate and ubiquitous mammals such as the common vole (*M*. *arvalis)* [122], which has been regularly identified in archaeological sites from the LGM in association with collared lemmings (*D*. *torquatus)* (e.g. [29,56]). Indeed the presence of *M*. *arvalis* remains have been retrieved in all the known Late Pleistocene archaeological sequences in southwestern France (Table 1). It is emphasized that this observation is at variance with the predictive distribution models based on the present-day distributions of European rodent species [108]. This spatial mosaic hypothesis of can also be proposed for the two forest-associated rodent species, *A*. *sylvaticus* and *E*. *quercinus*, which have few occurrences during the Late Pleistocene (Fig 6). In contrast with *M*. *arvalis*, these two rodent species had lower densities and probably more patchy distributions restricted to areas in which they were able to accommodate the LGM climatic conditions [123]. Moreover, this mosaic of refugia facilitates a rapid spread of species as soon as climatic conditions became favorable [105] (Fig 3).

In agreement with some previous studies (e.g. [14,18], the distribution patterns of rodent species both in space and time enforce the hypothesis of a strong climatic control on biodiversity at a regional scale. Indeed, the rapid fluctuations between warming and cold events that punctuated the last glacial period seem to have triggered both floral and faunal dynamics through successive geographic extensions and contractions of their populations.

## Concluding Remarks

This study has investigated the dynamics and evolution of rodent communities from southwestern France between 50 and 10 ky BP. The main result concerns the strong control of climatic changes on rodent biodiversity at both the regional and local scales. Other results of interest are as follows:

- The period between 50 and 19 ky BP reveals a relative stability in the structure of rodent communities, with little changes in species occurrences at a regional scale. The rapid climatic changes associated with the Heinrich and Dansgaard-Oeschger events, which punctuated the last glacial period, remained limited enough to preclude any complete renewal of the rodent community. However, these events seem to impact rodent populations at a local scale, extending or contracting populations in accordance to more or less favorable local climate conditions.
- Two major changes in the structure of rodent communities took place between 50 and 10 ky BP. The rodent communities from 50 to 19 ky were mainly composed of cold-climate species, which are no longer present in the region, while the latter ones, from 14.1 ky to present day, were dominated by temperate species that are still found today in southwestern France. The transition between these two types of communities occurred between the HE 1 and the Bølling events and was progressive with a succession of faunal changes concerning both temperate and cold-climate species.
- Small mammals survived within two types of refugia in southwestern France: in small areas that locally hosted favorable climatic conditions; and in mosaics of small populations scattered in multiple regions that have climatic conditions mostly unfavorable but not lethal for the considered species. Such refugia facilitate a rapid spread of species as soon as climatic conditions became favorable.

## Supporting Information

S1 Fig Square roots of the standard error of dates reported against the absolute date values (S1 Table). The line is the regression line obtained using the Reduced Major Axis method, which was also used to calculate the time-intervals reported in S2 Table. Grey circles illustrate the dates for Les Cottés (Vienne) that were excluded from the database used to perform the linear regression equation (see text “Building a time scale with increasing time-intervals”). In the upper left corner, standard errors of dates are reported against the absolute dates.
(EPS)

S2 Fig Temporal variation of species occurrence in southwestern France during the Late Pleistocene with 95% confidence envelope (grey areas). Rodent species are the following: the garden dormouse (*Eliomys quercinus*), the fat dormouse (*Glis glis*), the wood mouse (*Apodemus sylvaticus*), the bank vole (*Clethrionomys glareolus*), the snow vole (*Chionomys nivalis*), the Mediterranean pine vole (*Microtus (Terricola) duodecimcostatus*), the pine vole (*Microtus (Terricola) subterraneus*) and the southern water vole (*Arvicola sapidus*), the root vole (*Microtus oeconomus*), the ground squirrel (*Spermophilus* sp.), the narrow-headed vole (*Microtus gregalis*) and the collared lemming (*Dicrostonyx torquatus*). Grey intervals indicate the four Heinrich events and the Younger Dryas event. Hatched areas indicate time-intervals with no data. See Fig 2 for abbreviations.
(EPS)

S1 Table Compilation of radiocarbon dates available for the considered archaeological sites. Dates are calibrated at 2σ (95.4%) using OxCal 4.2 and calibration curve IntCal13 [64]. The minimum and maximum values correspond respectively to the 2.5% and 97.5% boundaries of minimum and maximum calibrated age distribution.
(DOC)

S2 Table Time-intervals defined with their standard errors as well as the number of stratigraphic units that cross over them. (DOC)

S3 Table Number of stratigraphic units in which each species is present for each time-interval. (DOC)
