# Supplementary material for: Investigating the Influence of Climate Changes on Rodent Communities at a Regional-Scale (MIS 1-3, Southwestern France)
Source: PLoS One. 2016 Jan 20;11(1):e0145600. doi: 10.1371/journal.pone.0145600 (PMC4720448; doi:10.1371/journal.pone.0145600)
Supplement: S1 Table — Dates are calibrated at 2σ (95.4%) using OxCal 4.2 and calibration curve IntCal13 [64]. The minimum and maximum values correspond respectively to the 2.5% and 97.5% boundaries of minimum and maximum calibrated age distribution. (DOC) [file pone.0145600.s003.doc]

| **Site number of Figure 1** | **Site** | **Archaeological level** | **Minimal date** | | | **Maximal date** | | | **Number of dates** | **Minimal value** | **Maximal value** | **References** |
| --- | --- | --- | --- | --- | --- | --- | --- | --- | --- | --- | --- | --- |
|  |  |  | **Name** | **Date** | **Standard deviation** | **Name** | **Date** | **Standard deviation** |  |  |  |  |
| **9** | **Abeurador** | 5 central area | MC-2144 | 8740 | 90 | - | - | - | 1 | 9541 | 10148 | (Vaquer and Ruas, 2009) |
| **9** | **Abeurador** | 7 central area | AA-13083 | 9755 | 110 | AA-13084 | 9845 | 115 | 2 | 10734 | 11760 | (Vaquer and Ruas, 2009) |
| **9** | **Abeurador** | 8 central area | Gif-6746 | 10480 | 100 | Ky-951 | 11090 | 190 | 2 | 12063 | 13314 | (Vaquer and Ruas, 2009) |
| **9** | **Abeurador** | 10 central area | Ly-949 | 14735 | 80 | - | - | - | 1 | 17689 | 18155 | (Vaquer and Ruas, 2009) |
| **32** | **Abri Ragout** | B | GRN-4677 | 12890 | 140 | - | - | - | 1 | 14985 | 15881 | (Dujardin and Tymula, 2005) |
| **18** | **Borie del Rey** | Laborian | Ly-1401 | 10350 | 340 | Ly-1386 | 10400 | 230 | 3 | 11178 | 12916 | (Coulonges, 1963) |
| **3** | **Bourrouilla** | b2 | Gif-9986 | 12395 | 35 | Gif-10002 | 12260 | 120 | 2 | 14175 | 14810 | (Chauchat, 1999) |
| **3** | **Bourrouilla** | b3 | Gif-10255 | 12780 | 40 | - | - | - | 1 | 15082 | 15385 | (Chauchat, 1999) |
| **3** | **Bourrouilla** | c | Gif-10234 | 12700 | 40 | Gif-10254 | 13220 | 80 | 3 | 14935 | 16154 | (Chauchat, 1999) |
| **6** | **Bois du Cantet** | boyau 7 | Ly-2614 | 10920 | 160 | - | - | - | 1 | 12570 | 13103 | (Clot et al., 1984) |
| **6** | **Bois du Cantet** | Magdalenian level | Ly-1404 | 13060 | 430 | Ly-1403 | 13370 | 270 | 3 | 14160 | 16915 | (Clot et al., 1984) |
| **25** | **Castanet** | Aurignacian A/Aurignacian 1 | GifA-97312 | 34800 | 1100 | GifA-97313 | 35200 | 1100 | 2 | 36834 | 42132 | (Rigaud, 2000) |
| **16** | **Combe-Grenal** | C20 | - | 44000 | 4000 | - | - | - | 1 | 43325 | >50000 | (d'Errico et al., 2011) |
| **23** | **Grotte de Comarque** | - | Ly-2154 | 12710 | 200 | Ly-2355 | 13370 | 340 | 2 | 14236 | 17148 | (Evin et al., 1983) |
| **14** | **Roc de Combe** | C2 | OxA-1258 | 24500 | 400 | - | - | - | 1 | 27789 | 29418 | (Hedges et al., 1990) |
| **14** | **Roc de Combe** | C5 | OxA 1441 | 28500 | 700 | OxA-1259 | 32000 | 1000 | 2 | 31212 | 38711 | (Hedges et al., 1990) |
| **14** | **Roc de Combe** | C7 | OxA-1262 | 33400 | 1100 | OxA-1263 | 34800 | 1200 | 2 | 35363 | 41903 | (Hedges et al., 1990) |
| **14** | **Roc de Combe** | C8 | GifA-101264 | 39540 | 970 | GifA-101265 | 45100 | 2100 | 1 | 42082 | >50000 | (Hedges et al., 1990) |
| **8** | **Conques** | C2 | - | 13330 | 140 | - | - | - | 1 | 15618 | 16465 | (Baills et al., 2003) |
| **8** | **Conques** | C3 | - | 14320 | 140 | - | - | - | 1 | 17055 | 17846 | (Baills et al., 2003) |
| **35** | **Les Cottés** | US 2 | S-eva 9717 | 31750 | 280 | S-eva 9719 | 32670 | 120 | 3 | 35027 | 36977 | (Talamo et al., 2012) |
| **35** | **Les Cottés** | US 4 upper phase | S-eva 9711 | 33180 | 160 | S-eva 9720 | 33860 | 160 | 2 | 36703 | 38752 | (Talamo et al., 2012) |
| **35** | **Les Cottés** | US 4 lower phase | S-eva 13672 | 34080 | 250 | S-eva 9713 | 35150 | 280 | 3 | 37960 | 40396 | (Talamo et al., 2012) |
| **35** | **Les Cottés** | US 6 | S-eva 13666 | 36230 | 210 | S-eva 9695 | 38540 | 270 | 4 | 40352 | 42972 | (Talamo et al., 2012) |
| **26** | **Combe-Saunière I** | III | Ly-3202 | 13910 | 230 | OxA-410 | 15750 | 230 | 7 | 16215 | 19590 | (Terberger and Street, 2002) |
| **26** | **Combe-Saunière I** | IV | OxA-489 | 19450 | 330 | OxA-753 | 19630 | 320 | 3 | 22636 | 24407 | (Terberger and Street, 2002) |
| **26** | **Combe-Saunière I** | VA | OxA-758 | 21640 | 400 | Ly-3330 | 21940 | 350 | 2 | 25154 | 27097 | (Gowlett et al., 1986) |
| **1** | **Dufaure** | 3 | ly-4223 | 10310 | 270 | - | - | - | 1 | 11271 | 12692 | (Straus, 1995) |
| **1** | **Dufaure** | 4 | ly 3182 | 12260 | 400 | Poz15985 | 12800 | 60 | 4 | 13394 | 15525 | (Straus, 1995) |
| **1** | **Dufaure** | 5 | ly -3591 | 12690 | 230 | ly-3923 | 12990 | 220 | 2 | 14154 | 16230 | (Straus, 1995) |
| **1** | **Dufaure** | 6 | LY-3582 | 14020 | 340 | AA 3029 | 14640 | 230 | 4 | 16110 | 18390 | (Straus, 1995) |
| **2** | **La grotte Duruthy à Sordes-L'Abbaye** | C3 | Ly-858 | 11150 | 220 | - | - | - | 1 | 12683 | 13432 | (Délibrias and Evin, 1980) |
| **2** | **La grotte Duruthy à Sordes-L'Abbaye** | C4 | Ly-859 | 13510 | 220 | Ly-860 | 13840 | 210 | 2 | 15683 | 17402 | (Délibrias and Evin, 1980) |
| **2** | **La grotte Duruthy à Sordes-L'Abbaye** | C5 | Ly-861 | 14180 | 200 | - | - | - | 1 | 16640 | 17806 | (Délibrias and Evin, 1980) |
| **5** | **Espélugues** | - | Ly-1406 | 13170 | 260 | - | - | - | 1 | 15064 | 16619 | (Clot and Omnès, 1979) |
| **24** | **Abri du Facteur** | Aurignacian | Gif-67 | 27890 | 2000 | - | - | - | 1 | 28479 | 38635 | (Delporte, 1968) |
| **24** | **Abri du Facteur** | Perigordian V | OxA-595 | 24210 | 500 |  | 25630 | 650 | 6 | 27502 | 31033 | (Djindjian, 2000) |
| **21** | **La Ferrassie** | D | Gif-2699 | 22520 | 500 | OxA-402 | 27900 | 770 | 5 | 25910 | 33721 | (d'Errico et al., 2011) |
| **17** | **Fieux** | F1C | Gif-6304 | 23900 | 330 | - | - | - | 1 | 27507 | 28653 | (Délibrias et al., 1987) |
| **19** | **Le Flageolet 1** | I-III | Ly-2185 | 18610 | 440 | Ly-1606 | 22440 | 680 | 2 | 21479 | 28000 | (Evin et al., 1985) |
| **19** | **Le Flageolet 1** | IV | Ly-1607 | 21190 | 920 | OxA-596 | 23250 | 500 | 3 | 23565 | 28476 | (Mellars et al., 1987) |
| **19** | **Le Flageolet 1** | V | Ly-2721 | 22520 | 500 | OxA-447 | 25700 | 700 | 2 | 25910 | 31131 | (Evin et al., 1985); (Mellars et al., 1987) |
| **19** | **Le Flageolet 1** | VII | Ly-2723 | 25150 | 600 | Ly-2723 | 26150 | 600 | 3 | 28121 | 31259 | (Evin et al., 1985) |
| **19** | **Le Flageolet 1** | VIII-IX | Ly-2724 | 26800 | 1000 | Ly-2725 | 27350 | 1400 | 3 | 29005 | 34879 | (Evin et al., 1985) |
| **19** | **Le Flageolet 1** | XI | GifA-95538 | 32040 | 850 | GifA-95559 | 34300 | 1100 | 3 | 34532 | 41340 | (Rigaud, 2000) |
| **19** | **Le Flageolet 2** | II | Ly-916 | 12870 | 390 | - | - | - | 1 | 14030 | 16499 | (Délibrias and Evin, 1980) |
| **19** | **Le Flageolet 2** | IX | Ly-917 | 14110 | 690 | Ly-918 | 15250 | 320 | 3 | 15271 | 19241 | (Djindjian, 2003) |
| **11** | **Abri Fontalès** | - | GifA-96327 | 13140 | 120 | - | - | - | 1 | 15338 | 16115 | (Gambier et al., 2000) |
| **36** | **Abri Fritsch à Pouligny-Saint-Pierre** | - | Ly-1122 | 16530 | 550 | Ly-1124 | 17980 | 350 | 4 | 18766 | 22535 | (Délibrias and Evin, 1980) |
| **7** | **La vache** | "Salle Monique" | Gif-7603 | 12800 | 140 | GifA-96479 | 13770 | 140 | 4 | 14732 | 17086 | (Clottes and Delporte, 2003) |
| **34** | **Garenne (Grand Abri)** | B1/B2 | Ly-3000 | 14270 | 270 | Ly-1126 | 15560 | 580 | 2 | 16571 | 20335 | (Délibrias and Evin, 1980) |
| **4** | **Isturitz** | Late Magdalenian | OxA-19837 | 12245 | 60 | OxA-19835 | 13455 | 55 | 3 | 13962 | 16403 | (Szmidt et al., 2009) |
| **4** | **Isturitz** | Middle Magdalenian | OxA-19830 | 13910 | 70 | OxA-19836 | 15130 | 110 | 4 | 16564 | 18651 | (Szmidt et al., 2009) |
| **28** | **Roc de Marcamps** | 2 | Ly-2680 | 13570 | 420 | Ly-2291 | 14910 | 240 | 3 | 15216 | 18680 | (Evin et al., 1985) |
| **28** | **Roc de Marcamps** | 3 | Ly-4222 | 15070 | 270 | Ly-2681 | 15700 | 450 | 2 | 17705 | 20096 | (Djindjian, 2003) |
| **28** | **Roc de Marcamps** | 4 | Ly-4219 | 16840 | 520 | Ly-4220 | 17880 | 290 | 3 | 19147 | 22368 | (Djindjian, 2003) |
| **28** | **Roc de Marcamps** | 5 | Ly-4221 | 18290 | 330 | - | - | - | 1 | 21323 | 22916 | (Djindjian, 2003) |
| **28** | **Roc de Marcamps** | 8 | Ly-2682 | 26520 | 830 | - | - | - | 1 | 28920 | 32589 | (Evin et al., 1985) |
| **20** | **Moulin du Roc (Saint-Chamassy, Dordogne)** | "Couche Bigarrée" | - | 11340 | 170 | - | - | - | 1 | 12813 | 13534 | (Oppliger, 2008) |
| **20** | **Moulin du Roc (Saint-Chamassy, Dordogne)** | "Couche Brune S.I." | - | 12700 | 50 | - | 12810 | 60 | 2 | 14893 | 15540 | (Oppliger, 2008) |
| **20** | **Moulin du Roc (Saint-Chamassy, Dordogne)** | "Couche Jaune" | - | 15600 | 1200 | - | - | - | 1 | 16195 | 22419 | (Oppliger, 2008) |
| **22** | **Abri Pataud** | 3 (Perigordian VI) | GrN-1892 | 21540 | 160 | OxA-686 | 24500 | 600 | 9 | 25543 | 30075 | (d'Errico et al., 2011) |
| **22** | **Abri Pataud** | 5 (Perigordian IV) | Gx-1371 | 25815 | 330 | OxA-169 | 28400 | 1100 | 9 | 29275 | 34906 | (d'Errico et al., 2011) |
| **22** | **Abri Pataud** | 7/8 | GrN-3105 | 29300 | 450 | GrN-3116 | 33290 | 700 | 5 | 32280 | 39311 | (d'Errico et al., 2011) |
| **12** | **Peyrugues** | 14 | GifA-95474 | 21700 | 250 | - | - | - | 1 | 25517 | 26533 | (Allard et al., 2009) |
| **12** | **Peyrugues** | 12 | GifA-96225 | 19410 | 200 | GifA-95460 | 20910 | 220 | 2 | 22891 | 25700 | (Allard et al., 2009) |
| **12** | **Peyrugues** | 16 | Ly-3594(SacA-5532) | 17890 | 100 | Ly-3593 | 18910 | 110 | 3 | 21365 | 23059 | (Allard et al., 2009) |
| **12** | **Peyrugues** | 18 | GifA-92169 | 22400 | 280 | GifA-96224 | 22750 | 250 | 2 | 26085 | 27535 | (Allard et al., 2009) |
| **12** | **Peyrugues** | 20 | Ly-3596 | 23150 | 170 | Ly-3595(SacA-5533) | 23520 | 180 | 2 | 27142 | 27946 | (Allard et al., 2009) |
| **12** | **Peyrugues** | 22 | LY-3598 | 24200 | 190 | Gif-7998 | 24800 | 500 | 2 | 27839 | 30155 | (Allard et al., 2009) |
| **12** | **Peyrugues** | 3 | Gif-7592 | 13020 | 140 | Ly-3600(SacA-5538) | 13960 | 100 | 3 | 15186 | 17292 | (Allard et al., 2009) |
| **12** | **Peyrugues** | 5 | GifA-95450 | 15940 | 150 | GifA-95447 | 17660 | 160 | 4 | 18886 | 21819 | (Allard et al., 2009) |
| **12** | **Peyrugues** | 6 | GifA-96228 | 18600 | 140 | GifA-93084 | 18740 | 200 | 3 | 22146 | 23127 | (Allard et al., 2009) |
| **12** | **Peyrugues** | 9 | Gif-7996 | 18100 | 140 | - | - | - | 1 | 21570 | 22329 | (Allard et al., 2009) |
| **15** | **Le Piage** | F | Gif-5027 | 29000 | 1000 | GifA-101260 | 32800 | 700 | 2 | 31158 | 38744 | (Délibrias et al., 1987) |
| **30** | **Abri Pintaud-Gaudry** | 2 | BM-2309 | 14770 | 270 | - | - | - | 2 | 17347 | 18625 | (Dujardin and Tymula, 2005) |
| **24** | **Pont d'Ambon** | 2-3A | Gif-3740 | 9640 | 120 | GifA-99102 | 10730 | 100 | 3 | 10607 | 12805 | (Délibrias and Evin, 1980) |
| **24** | **Pont d'Ambon** | 3BC | Gif-7223 | 11600 | 120 | Gif-3739 | 12130 | 160 | 2 | 13206 | 14706 | (Délibrias and Evin, 1980) |
| **24** | **Pont d'Ambon** | 4 | Gif-3369 | 12840 | 220 | - | - | - | 1 | 14405 | 16017 | (Délibrias and Evin, 1980) |
| **13** | **Peyrazet** | C2 | Lyon-7828 | 9780 | 42 | - | - | - | 1 | 11151 | 11255 | Unpublished data |
| **13** | **Peyrazet** | C3 | Lyon-7826 | 11810 | 50 | - | - | - | 1 | 13485 | 13756 | Unpublished data |
| **13** | **Peyrazet** | C4sommet | Lyon-7827 | 12180 | 60 | - | - | - | 1 | 13835 | 14245 | Unpublished data |
| **13** | **Peyrazet** | us 7 | - | 12580 | 80 | - | 12720 | 80 | 2 | 14405 | 15409 | (Langlais et al., 2012) |
| **32** | **Bois Ragot** | 5 | OXA-12079 (LYON-2102) | 12560 | 50 | OXA-10331 (LYON-1370) | 12685 | 70 | 6 | 14529 | 15310 | (Chollet and Dujardin, 2005) |
| **29** | **La Roche à Pierrot** | EJJ |  | 32100 | 3000 | - | - | - | 1 | 31332 | 46557 | (Drucker et al., 1999) |
| **29** | **La Roche à Pierrot** | EJOP |  | 36300 | 2700 | - | - | - | 1 | 36156 | 48270 | (Dujardin and Tymula, 2005) |
| **10** | **Grotte du Salpêtre de Pompignan** | 1A | MC-2241 | 9900 | 200 | - | - | - | 1 | 10740 | 12097 | (d'Errico et al., 2011) |
| **33** | **Taillis des Coteaux** | IIG | Ly-3876 | 14630 | 75 | - | 15280 | 90 | 2 | 17596 | 18751 | (Primault et al., 2007) |
| **33** | **Taillis des Coteaux** | IIIA | Ly-2264 | 16920 | 170 | Ly-6406 | 17190 | 110 | 3 | 19992 | 21054 | (Primault et al., 2007) |
| **33** | **Taillis des Coteaux** | IIIB | Ly-6407 | 17460 | 110 | - | - | - | 1 | 20753 | 21446 | (Primault et al., 2007) |
| **33** | **Taillis des Coteaux** | Vd | Ly-2639 | 18140 | 145 | - | - | - | 1 | 21615 | 22364 | (Primault et al., 2007) |
| **33** | **Taillis des Coteaux** | VI A | - | 20870 | 85 | - | - | - | 1 | 24904 | 25504 | (Primault et al., 2007) |
| **33** | **Taillis des Coteaux** | VI G | - | 24950 | 135 | - | - | - | 1 | 28665 | 29370 | (Primault et al., 2007) |
| **33** | **Taillis des Coteaux** | VII A | Beta-210191 | 29840 | 210 | - | - | - | 1 | 33604 | 34345 | (Primault et al., 2007) |
|  |  |  |  |  |  |  |  |  |  |  |  |  |
|  |  |  |  |  |  |  |  |  |  |  |  |  |

**References :**

Allard, M., Juillard, F., Le Gall, O., Martin, H., and Jeannet, M. (2009). Faunes paléolithiques des Peyrugues à Orniac, Lot. *Préhistoire du Sud-Ouest* 17, 141-217.

Baills, H., avec la collaboration de, Moigne, A.-M., Grégoire, S., et les contributions de, Abdulffatah, S., André, J., Barsky, D. R., Batalla, G., Berlic, P., Calvet, M., Campmajo, P., Delibrias, G., Desclaux, E., Djerrab, A., Falguères, C., Fullola Pericot, J. M., Grillet, J.-L., Heinz, C., Hue, M., Lenoble, J.-L., Mokhtari, B., Oberlin, C., Pois, V., and Renault-Miskovsky, J. (2003). Les Conques : des chasseurs magdaléniens et leur territoire. Service de Préhistoire, Liège.

Chauchat, C. (1999). L'habitat Magdalénien de la grotte du Bourrouilla à Arancou (Pyrénées-Atlantiques). *Gallia Préhistoire* 41, 1-152.

Chollet, A., and Dujardin, V. (2005). La grotte du Bois-Ragot à Gouex (Vienne) : Magdalénien et Azilien : essais sur les hommes et leurs environnements. Société Préhistorique Française, Paris.

Clot, A., Brochet, G., Chaline, J., Desse, G., Evin, J., Granier, J., Mein, P., Mourer-Chauvire, C., Omnès, J., and Rage, J. C. (1984). Faune de la grotte préhistorique du bois de Cantet (Espèche, Hautes-Pyrénées, France). *Munibe* 36, 33-50.

Clot, A., and Omnès, J. (1979). Premiers datages radiocarbone du Magdalénien des Hautes-Pyrénées. *Bulletin de la Société Préhistorique Française* 76, 324-337.

Clottes, J., and Delporte, H. (2003). La grotte de La Vache (Ariège) : Fouilles Romain Robert. Volume 1: Les occupations du Magdalénien. CTHS / RMN, Paris.

Coulonges, L. (1963). Magdalénien et Périgordien post-glaciaires : la grotte de la Borie del Rey (Lot-et-Garonne). *Gallia Préhistoire* 6, 1-29.

d'Errico, F., Banks, W. E., Vanhaeren, M., Laroulandie, V., and Langlais, M. (2011). PACEA Geo-Referenced Radiocarbon Database. *PaleoAnthropology*, 1-12.

Délibrias, G., and Evin, J. (1980). Sommaire des datations ^14^C concernant la préhistoire en France: II. - Dates parues de 1974 à 1978. *Bulletin de la Société Préhistorique Française* 77, 215-224.

Délibrias, G., Guillier, M. T., Évin, J., and Chevallier, J. (1987). Sommaire des datations ^14^C concernant la préhistoire en France: III. Dates effectuées de 1979 à fin 1984. *Bulletin de la Société Préhistorique Française* 84, 207-223.

Delporte, H. (1968). L'abri du Facteur à Tursac. 1. Etude générale. *Gallia Préhistoire* 11, 1-112.

Djindjian, F. (2000). The mid upper Palaeolithic (30,000 to 20,000 BP) in France. *In:* W. Roebroeks, M. Mussi, J. Svoboda, and K. Fennema, Eds.)" Hunters of the Golden Age: The Mid Upper Palaeolithic of Eurasia 30,000 - 20,000 BP." University, Leiden, pp. 313-324.

Djindjian, F. (2003). Hypothèses de peuplement Paléolithique entre 18500 et 16000 BP en Aquitaine et en Languedoc. *Préhistoire du Sud-Ouest* Supplément n°6, 29-46.

Drucker, D., Bocherens, H., Mariotti, A., Lévêque, F., Vandermeersch, B., and Guadelli, J.-L. (1999). Conservation des signatures isotopiques du collagène d'os et de dents du Pléistocène supérieur (Saint-Césaire, France) : implications pour les reconstitutions des régimes alimentaires des Néandertaliens. *Bulletins et Mémoires de la Société d'Anthropologie de Paris* 11, 289-305.

Dujardin, V., and Tymula, S. (2005). Relecture chronologique de sites paléolithiques et épipaléolithiques anciennement fouillés en Poitou-Charentes. *Bulletin de la Société Préhistorique Française* 102, 771-788.

Evin, J., Marechal, J., and Marien, G. (1983). Lyon natural radiocarbon measurements IX. *Radiocarbon* 25, 59-128.

Evin, J., Marechal, J., and Marien, G. (1985). Lyon natural radiocarbon measurements X. *Radiocarbon* 27, 386-454.

Gambier, D., Valladas, H., Tisnerat, N., Arnold, M., and Bresson, F. (2000). Datation de vestiges humains présumés du Paléolithique supérieur par la méthode du Carbone 14 en spectrométrie de masse par accélérateur. *Paléo* 12, 201-212.

Gowlett, J. A. J., Hedges, R. E. M., Law, I. A., and Perry, C. (1986). Radiocarbon dates from the Oxford AMS system: Archaeometry datelist 4. *Archaeometry* 28, 206-221.

Hedges, R. E. M., Housley, R. A., Law, I. A., and Bronk Ramsey, C. (1990). Radiocarbon dates from the Oxford AMS system: Archaeometry datelist 10. *Archaeometry* 32, 101-108.

Langlais, M., Costamagno, S., Laroulandie, V., Pétillon, J.-M., Discamps, E., Mallye, J.-B., Cochard, D., and Kuntz, D. (2012). The evolution of Magdalenian societies in South-West France between 18,000 and 14,000 cal BP: Changing environments, changing tool kits. *Quaternary International* 272-273, 138-149.

Mellars, P. A., Bricker, H. M., and Gowlett, A. J. (1987). Radiocarbon accelerator dating of French Upper Palaeolithic sites. *Current Anthropology* 28, 129-133.

Oppliger, J. (2008). Les micromammifères (*Chiroptera*, *Insectivora* et *Rodentia*) comme indicateurs de l'environnement au Tardiglaciaire et à l'Holocène : le cas du Moulin du Roc (Saint-Chamassy, Dordogne, France). Unpublished Travail de diplôme (tutorat scientifique : Marcel Jeannet) thesis, Département d’Anthropologie et d’Ecologie de l’Université de Genève.

Primault, J., Brou, L., Gabilleau, J., Langlais, M., avec la collaboration de, Berthet, A.-L., Griggo, C., Guérin, S., Gambier, D., Houmard, C., Laroulandie, V., Le Brun-Ricalens, F., Liard, M., Liolios, D., Mistrot, V., Rambaud, D., Schmitt, A., Soler, L., Taborin, Y., and Vissac, C. (2007). La grotte du Taillis des Coteaux à Antigny (Vienne) : intérêts d'une séquence originale à la structuration des premiers temps du Magdalénien. *Bulletin de la Société Préhistorique Française* 104, 743-758.

Rigaud, J.-P. (2000). Human adaptations to the climatic deterioration of the last Pleniglacial in southwestern France (30,000-20,000 BP). *In:* W. Roebroeks, M. Mussi, J. Svoboda, and K. Fennema, Eds.)" Hunters of the golden age." Leiden, pp. 325-336.

Straus, L. G. (1995). Les derniers chasseurs de rennes du monde pyrénéen. L'abri Dufaure : un gisement tardiglaciaire en Gascogne. Société Préhistorique Française, Paris.

Szmidt, C. C., Pétillon, J.-M., Cattelain, P., Normand, C., and Schwab, C. (2009). Premières dates radiocarbone pour le Magdalénien d'Isturitz (Pyrénées-Atlantiques). *Bulletin de la Société Préhistorique Française* 106, 588-592.

Talamo, S., Soressi, M., Roussel, M., Richards, M., and Hublin, J.-J. (2012). A radiocarbon chronology for the complete Middle to Upper Palaeolithic transitional sequence of Les Cottés (France). *Journal of Archaeological Science* 39, 175-183.

Terberger, T., and Street, M. (2002). Hiatus or continuity ? New results for the question of pleniglacial settlement in Central Europe. *Antiquity* 76, 691-698.

Vaquer, J., and Ruas, M.-P. (2009). La grotte de l'Abeurador Félines-Minervois (Hérault) : occupations humaines et environnement du Tardiglaciaire à l'Holocène. *In:* M. Barbaza, (Ed.) De Méditerranée et d'ailleurs ... Mélanges offerts à Jean Guilaine." Archives d'Ecologie Préhistorique, Toulouse, pp. 761-792.
