# Supplementary material for: Investigating the Influence of Climate Changes on Rodent Communities at a Regional-Scale (MIS 1-3, Southwestern France)
Source: PLoS One. 2016 Jan 20;11(1):e0145600. doi: 10.1371/journal.pone.0145600 (PMC4720448; doi:10.1371/journal.pone.0145600)
Supplement: S2 Table — (DOC) [file pone.0145600.s004.doc]

| **Time-intervals** | **Duration** | **Number of sites** |
| --- | --- | --- |
| 10000-10080 | 80 | 1 |
| 10080-10170 | 90 | 1 |
| 10170-10264 | 93 | 0 |
| 10264-10361 | 97 | 0 |
| 10361-10461 | 100 | 0 |
| 10461-10565 | 104 | 0 |
| 10565-10673 | 108 | 0 |
| 10673-10785 | 112 | 1 |
| 10785-10901 | 116 | 3 |
| 10901-11023 | 121 | 3 |
| 11023-11149 | 126 | 3 |
| 11149-11280 | 131 | 3 |
| 11280-11416 | 137 | 5 |
| 11416-11559 | 143 | 5 |
| 11559-11708 | 149 | 5 |
| 11708-11864 | 156 | 5 |
| 11864-12026 | 163 | 4 |
| 12026-12197 | 170 | 4 |
| 12197-12376 | 179 | 4 |
| 12376-12563 | 187 | 4 |
| 12563-12760 | 197 | 4 |
| 12760-12967 | 207 | 5 |
| 12967-13185 | 218 | 4 |
| 13185-13415 | 230 | 3 |
| 13415-13659 | 243 | 4 |
| 13659-13916 | 257 | 3 |
| 13916-14188 | 272 | 3 |
| 14188-14477 | 289 | 8 |
| 14477-14784 | 307 | 10 |
| 14784-15111 | 327 | 11 |
| 15111-15460 | 349 | 15 |
| 15460-15833 | 373 | 16 |
| 15833-16234 | 400 | 16 |
| 16234-16663 | 430 | 14 |
| 16663-17126 | 463 | 13 |
| 17126-17626 | 500 | 12 |
| 17626-18168 | 542 | 11 |
| 18168-18756 | 589 | 10 |
| 18756-19398 | 642 | 5 |
| 19398-20100 | 702 | 7 |
| 20100-20872 | 772 | 6 |
| 20872-21725 | 852 | 6 |
| 21725-22670 | 945 | 9 |
| 22670-23725 | 1055 | 5 |
| 23725-24908 | 1183 | 4 |
| 24908-26244 | 1336 | 4 |
| 26244-27765 | 1520 | 8 |
| 27765-29509 | 1744 | 9 |
| 29509-31529 | 2020 | 10 |
| 31529-33895 | 2365 | 8 |
| 33895-36699 | 2804 | 8 |
| 36699-40070 | 3372 | 9 |
| 40070-44194 | 4124 | 6 |
| 44194-49341 | 5147 | 4 |
| 49341-55923 | 6482 | 2 |
