# Supplementary material for: Investigating the Influence of Climate Changes on Rodent Communities at a Regional-Scale (MIS 1-3, Southwestern France)
Source: PLoS One. 2016 Jan 20;11(1):e0145600. doi: 10.1371/journal.pone.0145600 (PMC4720448; doi:10.1371/journal.pone.0145600)
Supplement: S3 Table — (DOC) [file pone.0145600.s005.doc]

| **Time-intervals** | ***Microtus arvalis/agrestis*** | ***Microtus oeconomus*** | ***Microtus gregalis*** | ***Microtus (T.) duodecimcostatus*** | ***Microtus (T.) multiplex*** | ***Microtus (T.) subterraneus*** | ***Clethrionomys glareolus*** | ***Spermophilus sp.*** | ***Dicrostonyx torquatus*** | ***Chionomys nivalis*** | ***Arvicola sapidus*** | ***Arvicola terrestris*** | ***Apodemus sylvaticus*** | ***Eliomys quercinus*** | ***Glis glis*** | ***Sicista betulina*** | ***Cricetus cricetus*** |  |
| --- | --- | --- | --- | --- | --- | --- | --- | --- | --- | --- | --- | --- | --- | --- | --- | --- | --- | --- |
| **10785-10901** | 3 | 2 | 2 | 2 | 0 | 1 | 2 | 1 | 0 | 2 | 0 | 3 | 3 | 3 | 2 | 0 | 0 |  |
| **10901-11023** | 3 | 2 | 2 | 2 | 0 | 1 | 2 | 1 | 0 | 2 | 0 | 3 | 3 | 3 | 2 | 0 | 0 |  |
| **11023-11149** | 3 | 2 | 2 | 2 | 0 | 1 | 2 | 1 | 0 | 2 | 0 | 3 | 3 | 3 | 2 | 0 | 0 |  |
| **11149-11280** | 3 | 2 | 2 | 2 | 0 | 1 | 2 | 1 | 0 | 2 | 0 | 3 | 3 | 3 | 2 | 0 | 0 |  |
| **11280-11416** | 5 | 3 | 2 | 2 | 0 | 1 | 2 | 1 | 0 | 3 | 0 | 5 | 4 | 3 | 2 | 0 | 0 |  |
| **11416-11559** | 5 | 3 | 2 | 2 | 0 | 1 | 2 | 1 | 0 | 3 | 0 | 5 | 4 | 3 | 2 | 0 | 0 |  |
| **11559-11708** | 5 | 3 | 2 | 2 | 0 | 1 | 2 | 1 | 0 | 3 | 0 | 5 | 4 | 3 | 2 | 0 | 0 |  |
| **11708-11864** | 5 | 3 | 2 | 2 | 0 | 1 | 2 | 1 | 0 | 3 | 0 | 5 | 4 | 3 | 2 | 0 | 0 |  |
| **11864-12026** | 4 | 2 | 2 | 1 | 0 | 1 | 2 | 1 | 0 | 2 | 0 | 4 | 3 | 2 | 1 | 0 | 0 |  |
| **12026-12197** | 4 | 2 | 2 | 1 | 0 | 1 | 2 | 1 | 0 | 2 | 0 | 4 | 3 | 2 | 1 | 0 | 0 |  |
| **12197-12376** | 4 | 3 | 1 | 1 | 0 | 1 | 2 | 1 | 0 | 2 | 0 | 4 | 3 | 2 | 1 | 0 | 0 |  |
| **12376-12563** | 4 | 3 | 1 | 1 | 0 | 1 | 2 | 1 | 0 | 2 | 0 | 4 | 3 | 2 | 1 | 0 | 0 |  |
| **12563-12760** | 4 | 3 | 1 | 1 | 0 | 1 | 2 | 1 | 0 | 2 | 0 | 4 | 3 | 2 | 1 | 0 | 0 |  |
| **12760-12967** | 5 | 3 | 1 | 1 | 0 | 2 | 3 | 1 | 0 | 2 | 0 | 4 | 4 | 3 | 2 | 0 | 0 |  |
| **12967-13185** | 4 | 3 | 0 | 1 | 0 | 2 | 2 | 0 | 0 | 1 | 0 | 3 | 4 | 2 | 3 | 0 | 0 |  |
| **13185-13415** | 3 | 2 | 0 | 1 | 0 | 1 | 1 | 0 | 0 | 1 | 0 | 3 | 3 | 1 | 2 | 0 | 0 |  |
| **13415-13659** | 4 | 3 | 1 | 0 | 0 | 0 | 1 | 0 | 0 | 0 | 0 | 4 | 4 | 1 | 1 | 0 | 0 |  |
| **13659-13916** | 3 | 2 | 1 | 0 | 0 | 0 | 2 | 0 | 0 | 0 | 0 | 3 | 2 | 2 | 0 | 1 | 0 |  |
| **13916-14188** | 3 | 3 | 1 | 0 | 0 | 0 | 2 | 0 | 0 | 0 | 0 | 3 | 3 | 2 | 0 | 1 | 0 |  |
| **14188-14477** | 8 | 6 | 1 | 0 | 0 | 2 | 3 | 0 | 0 | 0 | 1 | 8 | 7 | 2 | 0 | 1 | 0 |  |
| **14477-14784** | 10 | 8 | 2 | 0 | 0 | 2 | 3 | 1 | 1 | 1 | 1 | 10 | 7 | 3 | 0 | 1 | 0 |  |
| **14784-15111** | 11 | 8 | 2 | 0 | 0 | 3 | 3 | 3 | 2 | 1 | 1 | 11 | 8 | 4 | 0 | 1 | 0 |  |
| **15111-15460** | 15 | 10 | 4 | 0 | 0 | 5 | 4 | 4 | 2 | 2 | 2 | 14 | 10 | 5 | 1 | 1 | 0 |  |
| **15460-15833** | 16 | 11 | 6 | 0 | 0 | 4 | 2 | 4 | 2 | 4 | 2 | 15 | 12 | 7 | 2 | 0 | 0 |  |
| **15833-16234** | 16 | 9 | 5 | 0 | 0 | 3 | 1 | 5 | 2 | 5 | 2 | 14 | 11 | 7 | 1 | 0 | 0 |  |
| **16234-16663** | 14 | 8 | 4 | 0 | 0 | 2 | 1 | 4 | 1 | 5 | 2 | 12 | 10 | 7 | 1 | 0 | 0 |  |
| **16663-17126** | 13 | 10 | 5 | 0 | 0 | 1 | 1 | 4 | 2 | 4 | 0 | 13 | 6 | 6 | 1 | 0 | 0 |  |
| **17126-17626** | 12 | 10 | 5 | 0 | 0 | 1 | 0 | 4 | 2 | 5 | 1 | 12 | 5 | 6 | 1 | 0 | 1 |  |
| **17626-18168** | 11 | 10 | 7 | 0 | 0 | 2 | 2 | 6 | 3 | 5 | 1 | 11 | 5 | 6 | 1 | 0 | 1 |  |
| **18168-18756** | 10 | 10 | 8 | 0 | 0 | 2 | 2 | 6 | 3 | 4 | 0 | 10 | 4 | 5 | 1 | 0 | 1 |  |
| **18756-19398** | 5 | 5 | 5 | 0 | 0 | 1 | 0 | 3 | 1 | 3 | 0 | 5 | 1 | 2 | 1 | 0 | 0 |  |
| **19398-20100** | 7 | 7 | 6 | 0 | 0 | 0 | 0 | 4 | 2 | 3 | 1 | 6 | 2 | 2 | 0 | 0 | 0 |  |
| **20100-20872** | 6 | 6 | 5 | 0 | 1 | 0 | 1 | 3 | 3 | 3 | 2 | 5 | 2 | 2 | 0 | 0 | 0 |  |
| **20872-21725** | 6 | 6 | 5 | 0 | 1 | 0 | 1 | 3 | 3 | 2 | 3 | 5 | 2 | 2 | 0 | 0 | 0 |  |
| **21725-22670** | 9 | 8 | 6 | 0 | 0 | 0 | 0 | 3 | 3 | 3 | 2 | 8 | 2 | 2 | 0 | 0 | 0 |  |
| **22670-23725** | 5 | 4 | 3 | 0 | 0 | 0 | 0 | 2 | 2 | 2 | 0 | 5 | 1 | 1 | 0 | 0 | 0 |  |
| **23725-24908** | 4 | 4 | 3 | 0 | 0 | 0 | 0 | 1 | 2 | 2 | 1 | 4 | 2 | 2 | 0 | 0 | 0 |  |
| **24908-26244** | 4 | 4 | 3 | 0 | 0 | 0 | 0 | 1 | 2 | 1 | 1 | 4 | 2 | 1 | 0 | 0 | 0 |  |
| **26244-27765** | 8 | 5 | 5 | 0 | 0 | 0 | 0 | 0 | 1 | 2 | 1 | 8 | 2 | 3 | 0 | 0 | 0 |  |
| **27765-29509** | 9 | 4 | 4 | 0 | 1 | 0 | 1 | 0 | 1 | 3 | 2 | 9 | 2 | 2 | 0 | 0 | 0 |  |
| **29509-31529** | 10 | 2 | 5 | 0 | 0 | 0 | 2 | 2 | 3 | 5 | 0 | 10 | 1 | 3 | 0 | 0 | 0 |  |
| **31529-33895** | 8 | 3 | 6 | 0 | 0 | 0 | 0 | 1 | 1 | 3 | 0 | 8 | 1 | 2 | 0 | 0 | 0 |  |
| **33895-36699** | 8 | 3 | 6 | 0 | 0 | 1 | 0 | 2 | 1 | 3 | 0 | 8 | 3 | 4 | 0 | 0 | 0 |  |
| **36699-40070** | 9 | 3 | 8 | 0 | 0 | 1 | 0 | 1 | 1 | 4 | 0 | 8 | 1 | 4 | 0 | 0 | 0 |  |
| **40070-44194** | 6 | 2 | 5 | 0 | 0 | 0 | 0 | 3 | 0 | 2 | 0 | 6 | 0 | 2 | 0 | 0 | 0 |  |
| **44194-49341** | 4 | 1 | 3 | 0 | 0 | 0 | 0 | 1 | 0 | 2 | 0 | 4 | 1 | 1 | 0 | 0 | 0 |  |
| **49341-55923** | 2 | 1 | 1 | 0 | 0 | 0 | 0 | 1 | 0 | 2 | 0 | 2 | 1 | 1 | 0 | 0 | 0 |  |
|  |  |  |  |  |  |  |  |  |  |  |  |  |  |  |  |  |  |  |
